# Pluripotent Stem Cells for Disease Modeling and Drug Discovery in Niemann-Pick Type C1

**DOI:** 10.3390/ijms22020710

**Published:** 2021-01-12

**Authors:** Christin Völkner, Maik Liedtke, Andreas Hermann, Moritz J. Frech

**Affiliations:** 1 Translational Neurodegeneration Section “Albrecht Kossel”, Department of Neurology, University Medical Center Rostock, 18147 Rostock, Germany; christin.voelkner@med.uni-rostock.de (C.V.); maik.liedtke@med.uni-rostock.de (M.L.); andreas.hermann@med.uni-rostock.de (A.H.); 2 Center for Transdisciplinary Neurosciences Rostock (CTNR), University Medical Center Rostock, 18147 Rostock, Germany; 3 German Center for Neurodegenerative Diseases (DZNE) Rostock/Greifswald, 18147 Rostock, Germany

**Keywords:** induced pluripotent stem cells, iPSCs, patient-specific iPSCs, lysosomal storage disorders, NPC1, NPC2, cholesterol, neurodegeneration

## Abstract

The lysosomal storage disorders Niemann-Pick disease Type C1 (NPC1) and Type C2 (NPC2) are rare diseases caused by mutations in the *NPC1* or *NPC2* gene. Both NPC1 and NPC2 are proteins responsible for the exit of cholesterol from late endosomes and lysosomes (LE/LY). Consequently, mutations in one of the two proteins lead to the accumulation of unesterified cholesterol and glycosphingolipids in LE/LY, displaying a disease hallmark. A total of 95% of cases are due to a deficiency of NPC1 and only 5% are caused by NPC2 deficiency. Clinical manifestations include neurological symptoms and systemic symptoms, such as hepatosplenomegaly and pulmonary manifestations, the latter being particularly pronounced in NPC2 patients. NPC1 and NPC2 are rare diseases with the described neurovisceral clinical picture, but studies with human primary patient-derived neurons and hepatocytes are hardly feasible. Obviously, induced pluripotent stem cells (iPSCs) and their derivatives are an excellent alternative for indispensable studies with these affected cell types to study the multisystemic disease NPC1. Here, we present a review focusing on studies that have used iPSCs for disease modeling and drug discovery in NPC1 and draw a comparison to commonly used NPC1 models.

## 1. Introduction

Niemann-Pick disease type C is a rare monogenic neurovisceral lysosomal storage disorder, inherited in an autosomal recessive manner, with an estimated incidence of 1/120,000 [1]. Homozygous or compound heterozygous mutations in the *NPC1* (95%; OMIM # 257220) or *NPC2* (5%, OMIM # 607625) gene lead to impaired intracellular transport of cholesterol and glycosphingolipids, resulting in the accumulation of these lipids in late endosomes/lysosomes (LE/LY). Currently, 549 mutations in *NPC1* and 29 mutations in *NPC2* have been described [2]. The location of observed mutations is not limited to the cholesterol binding site; rather, they can be found throughout the whole sequence and can lead to misfolded protein, resulting in proteasomal degradation and hampered trafficking to the lysosome and therefore reduced lipid turnover. Clinical manifestations of patients do not show a strong genotype-phenotype correlation, but rather Niemann-Pick disease type C is characterized by heterogeneous phenotypic expression. Therefore, it is hardly possible to predict the clinical outcome caused by a specific mutation, suggesting that several factors may be involved in the pathogenesis of the disease. 

The clinical spectrum of NPC1 includes visceral manifestations, such as hepatosplenomegaly, and neurological symptoms, such as hypotonia, loss of motor skills, ataxia, seizures, dysphagia, dysarthria, supranuclear gaze palsy (VSGP), and dementia, as well as psychiatric symptoms. Systemic and neurological symptoms occur at different times, with systemic symptoms, which may be absent in 10–15% of cases, preceding neurological symptoms. The age of onset of symptoms defines the classification into perinatal, infantile (early and late), juvenile, and adolescent/adult forms of NPC1. The perinatal presentation includes patients up to three months of age and patients usually suffer from liver disease, including fetal ascites or fetal hydrops and prolonged neonatal cholestatic jaundice with progressive hepatosplenomegaly. Patients with the early infantile form, from three months to two years of age, may present with hepatosplenomegaly and show delayed motor developmental and central hypotonia, while VSGP is usually not recognized. Patients with the late infantile form (2–6 years) lose already acquired motor skills, resulting in frequent falling and clumsiness. They also show progressive ataxia, dystonia, dysphagia, and dysarthria. These patients die between the ages of 7 and 12 years. The juvenile presentation (6–15 years) may be accompanied by hepatosplenomegaly for years, and patients show poor school performance and impaired fine movements and later progressive ataxia and dysarthria, as well as dystonia, dysphagia, and cataplexy. VSGP is usually present. Affected patients die in their teens or second decade of life. The adolescent/adult form (>15 years) is described as the attenuated juvenile form and is often associated with psychiatric symptoms such as psychosis and depression (for review refer to [1,3]). 

Since the identification of the primary genetic defect in 1997 [4], substantial progress in understanding the pathophysiology of NPC1 has been made, but still the mechanisms underlying the constitution and progression of the disease are not exactly understood and a cure for NPC1 remains elusive. In addition, the rare occurrence of NPC1 is a major hurdle that hinders rapid progress, as disease diagnosis and the establishment of appropriate test populations for clinical trials are challenging.

Human primary cultures would allow us to study pathophysiological mechanisms, but this possibility is limited by the availability and accessibility of disease-affected tissues, such as liver and brain. Here, pluripotent stem cells offer an excellent alternative as they can be differentiated into specific disease-affected cell types. Induced pluripotent stem cell (iPSC) technology has been widely used to model lysosomal storage disorders including Gaucher’s disease, Pompe disease, Fabry disease, metachromatic leukodystrophy, the neuronal ceroid lipofuscinoses, several of the mucopolysaccharidoses and Niemann-Pick disease types A and C [5,6,7,8,9,10,11].

Research using iPSC-based model systems is progressing rapidly, and therefore we want to recapitulate the current status of iPSC-based model systems used to study the pathomechanisms of Niemann-Pick type C1 disease. To this end, we will travel from standard NPC1 models to the exciting field of pluripotent stem cells and finally discuss their application in disease modeling and drug discovery for NPC1.

## 2. Commonly Used NPC1 Model Systems

Most of the studies designed to investigate the pathophysiological features of NPC disease use in vitro cell culture models containing fibroblasts or animal models (for review, see also [12,13]). Fibroblasts can be easily obtained from skin biopsies. Nevertheless, skin biopsies are related to ethical issues, especially when taken from underaged patients. They are inexpensive and are widely commercially available via biorepositories and can be expanded for a limited number of passages, generating a solid research basis. The use of tissues severely affected by NPC1, namely brain and liver, is challenging in terms of their availability. There have been studies analyzing these tissues post-mortem [14,15]; however, the significance of the results is limited as they only represent advanced stages of the disease. 

The *NPC1* gene is highly conserved among eukaryotes, making it possible to generate NPC1 models ranging from mammals to fungi, including cat, mouse, zebrafish, fruit fly, nematode and yeast models [12]. A frequently used animal model is the Npc1^nih^ mouse model that arose as a spontaneous mutation in the BALB/c mouse strain, resulting in an NPC1 null allele [16]. This model shows clinical and biochemical features of NPC1. It is characterized by progressive weight loss, loss of motor coordination, ataxia, tremors, hindlimb paralysis, and decreased lifespan [17,18]. Additionally, one can observe axonal swelling, demyelination, gliosis, functional alterations of Purkinje cells [19,20], and as a major hallmark of NPC1, the loss of cerebellar Purkinje cells [21,22,23]. Another mouse model is the Npc1^spm^ mouse that arose spontaneously on the C57BL/Ks background and shows attenuated sphingomyelinase activity and an excess of sphingomyelin accumulation [24]. This is based on a point mutation that leads to a null allele. As such null NPC1 mice display an extreme phenotype, they only partially reflect the human NPC1 phenotype as very few NPC1 patients are homozygous for truncating mutations but most of the pathogenic alleles are point mutations leading to misfolded NPC1 protein. Further mouse models (for review, refer to [12]) carrying different mutations are available and are of great value for modeling NPC1, as they have helped us to decipher many mechanistic processes [25,26,27]. Common features of the various murine NPC1 models are the progressive loss of cerebellar Purkinje cells and astrogliosis, both of which can be observed in NPC1 patients, accompanied by progressive loss of body weight and a shortened lifespan. However, they do not represent complex human phenotypes, but can only reflect partial aspects of them. For example, NPC1 mice do not form neurofibrillary tangles or plaques from tau and β-amyloid proteins, which do form in human mutant NPC1 neurons and are discussed to be involved in neurodegeneration observed in NPC1. Additionally, the apolipoprotein E ε4 homozygosity that was found in humans [28] is missing in mice. As the use of animal models is expensive and time consuming, they are inadequate for high throughput drug screenings [29]. However, their value remains in preclinical testing for the most promising hits from large-scale screenings. As an alternative to murine model systems, human pluripotent stem cells, as well as specific neuronal and hepatic differentiation protocols, can be used to generate NPC1 disease-affected cell types. In the following, we give an overview of studies in which human iPSC-based model systems were used for NPC1 disease modeling and discuss these studies in context with commonly used models, such as fibroblasts or murine NPC1 models.

## 3. NPC1 Disease Modeling Using Pluripotent Stem Cells

Since the advent of iPSC technology, iPSC-based models, displaying disease relevant phenotypes, are indispensable for disease modeling and drug discovery (for review, refer to [30,31]). This holds especially true for rare diseases such as NPC1. Currently, to the best knowledge of the authors, 17 studies are available [11,20,32,33,34,35,36,37,38,39,40,41,42,43,44,45,46] investigating NPC1 and only one publication describes an NPC2 model [47], by means of human pluripotent stem cell-based in vitro models. Out of these 17 publications dealing with NPC1, 13 publications comprise iPSC-based NPC1 models, used for disease modeling and drug discovery. Four publications are methodological or technical reports focusing on the generation of iPSCs, without further studies on the pathophysiology of NPC1 [40,43,44,46]. One publication describes the use of human embryonic stem cells combined with the silencing of *NPC1* [32], and the remaining 16 studies are based on iPS cells. 

**Figure 1:**
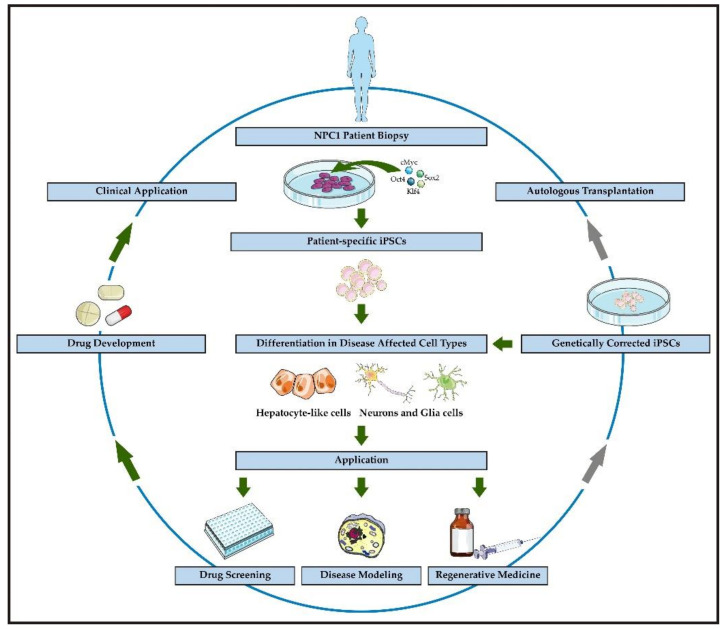
Schematic representation of the approaches using induced pluripotent stem cells (iPSCs) in the field of Niemann-Pick disease Type C1 (NPC1). Several studies describe the reprogramming of NPC1 patient-specific fibroblasts into disease-specific iPSCs, usually using the classical "Yamanaka factors". The majority of studies used neuronal cell systems derived from iPSCs. Currently, only two studies include hepatocyte-like cells derived from iPSCs. NPC1-deficient iPSC-derived cell types have been successfully used for disease modeling and drug discovery. Approaches to transplant genetically corrected cells have not yet been undertaken, but iPSCs with a TALEN-mediated correction of the *NPC1* mutation have been used for disease modeling. Green arrows indicate successful technical approaches, grey arrows indicate theoretical curative approaches. (Graphic was composed using https://smart.servier.com). Please also refer to Table 1.

Another two publications used multipotent adult stem cells [48] or neurons derived from the direct conversion of fibroblasts into neural stem cells [49]. The majority of the studies that focused on pathophysiological features of NPC1 have been performed with neuronal differentiated cells (NDCs), and only two studies included hepatocyte-like cells (HLCs) [33,36]. An overview of the publications is given in Table 1 and an overview of applications is given in Figure 1.

NPC1-associated phenotypes have been described in disease-affected cell types such as fibroblasts or animal models, whereas others were identified for the first time in iPSC-based models. Pathophysiological features of NPC1, using a human stem cell-based neuronal cell system, were first described by Ordonez and colleagues using human embryonic stem cells with an NPC1 knock down created by shRNA mediated silencing of NPC1 [32]. This study was followed by a report from Bergamin and colleagues [48], who isolated multipotent adult stem cells from skin biopsies (hSKIN-MASCs) of three NPC1 patients, thereby allowing the analysis of the impact of specific mutations and overcoming the ethical controversies of using human embryonic stem cells. Since then, several groups have described NPC1 model systems using patient-specific iPSCs, generated with protocols based on transduction by retrovirus, lentivirus and sendai virus. The first iPSC-based NPC1 model was described by our group in 2013 [11]. Below, we outline the results obtained with these NPC1 model systems, focused on pathophysiological features such as cholesterol accumulation, autophagy, mitochondrial homeostasis, oxidative stress, gliosis and functional alterations. An overview of the publications is given in Table 1. A comprehensive review in regards to pathophysiological features obtained from other NPC1 model systems can be found in [50].

The table contains an overview of publications involving the use or production of pluripotent stem cells in the field of NPC1 research. The corresponding references are given in square brackets.

### 3.1. Lipid Accumulation

The hallmark of NPC1, as well as of NPC2, is the accumulation of cholesterol and sphingolipids in late endosomes and lysosomes. For clinical diagnosis, filipin is routinely used to detect cholesterol accumulation in patient-derived fibroblasts. Filipin is a fluorescent antibiotic dye that specifically binds to unesterified cholesterol [51]. However, in patients with a mild clinical phenotype or in patients with a biochemical variant phenotype, in whom cholesterol accumulation cannot be detected despite clinical symptoms [1,52,53], filipin staining of cholesterol accumulation may fail.

Cholesterol accumulation has been shown in NPC1-deficient fibroblasts, iPSCs, neural progenitor cells (NPCs) /neural stem cells (NSCs) and neuronal differentiated cells (NDCs) [11,34,49], as well as in hepatocyte-like cells [33,36]. An accumulation can be recognized as a punctate pattern of a bright blue staining in the perinuclear region of the cells [54], but also in ramifications of neuronal differentiated cells [39]. The accumulation of cholesterol can be quantified using the Amplex Red Cholesterol assay, detecting the amount of total cholesterol (unesterified and esterified) [11,34,39,41].

The effect of cholesterol sequestration on downstream pathways, such as cholesterol ester synthesis, was investigated by Maetzel and colleagues [33]. By analyzing the incorporation of [^14^C]oleate, they observed a decreased cholesteryl [^14^C]oleate synthesis in NPC1-deficient cells compared to control cells. The authors suggested an impairment of cholesterol esterification in NPC1-deficient cells due to cholesterol sequestration [33]. One study lacked the proof of cholesterol accumulation in iPSC-derived neural stem cells and neurons [37]. It is still discussed to which extent neurons have the capacity to compensate for a cholesterol deficit by de novo synthesis compared to astrocytes, and to which extent neurons rely on astrocytes providing cholesterol [55,56]. Thus, the above-mentioned observation might be explained by a lack of an appropriate exogenous cholesterol source in the culture system and, indeed, several studies analyzing cholesterol accumulation in NPC1 used supplemented culture medium to provide an exogenous source of cholesterol [34,36].

In addition to the accumulation of cholesterol, deposits of GM2 and GM3 gangliosides are described in NPC1 patients´ brains [57] and fibroblasts [58], and in mouse brains [59]. GM2 accumulation has also been shown in human cellular in vitro model systems [11,48]. Bergamin and colleagues [48] described an accumulation of GM2 ganglioside in differentiated cells by immunostaining, which appears to be acquired during differentiation, as an accumulation in fibroblasts and undifferentiated hSKIN-MASCs was not detected. An accumulation of GM3 ganglioside in these cells was not observed. The authors concluded that the hSKIN-MASCs system might reflect an early stage of NPC1 disease, as a GM2 accumulation precedes the accumulation of GM3 [23]. Trilck and colleagues [39] showed a dotted staining pattern of accumulated GM2 in NPC1-deficient neurons. Colocalization analyses of GM2/βIII-tubulin and GM2/glial fibrillary acidic protein (GFAP) revealed a distribution of GM2 primarily in neurons. Comparable results were obtained from an NPC1 mouse model describing a lack of GM2 staining in astrocytes. As the GM2 staining colocalized with filipin staining, the authors suggested a deposit in the same cellular compartment [39]. Quantification of GM2 by HPLC-MS/MS revealed a four-fold higher amount of GM2 in two of the three analyzed mutants. GM2 degradation enzyme Hexosaminidase A (Hex A) was shown to have reduced activity. Comparable to the results of Bergamin and colleagues [48], Trilck and colleagues also failed to detect GM3 enrichment.

Taken together, cholesterol accumulation was shown in a variety of different cell types and patient-specific iPSCs or iPSC-derived cells. Nevertheless, it is difficult to compare the enrichments in the different cell systems because they depend on the protocols used for cell culture, e.g., in regard to a supplementary cholesterol source. However, the studies summarized here show not only the ubiquitous defect caused by mutations in the *NPC1* gene, but also the applicability of iPSC-based model systems for disease modeling of NPC1, at least in regard to the accumulation of different lipids.

### 3.2. Alterations of Autophagy

Autophagy is a physiological catabolic mechanism that provides cellular material for degradation and recycling in the lysosome. This enables the rapid elimination of toxic waste, misfolded proteins or damaged organelles in pathophysiological situations, but also the reuse of material no longer required under physiological conditions [60]. Thus, autophagy contributes to cellular stress response pathways, which are especially important in long-lived, post-mitotic cells that cannot dilute damaged material via cell division, such as neurons [60]. During autophagy, the material to be degraded is engulfed in double-membranous structures called autophagosomes. These organelles undergo different fusion events with endosomes, forming so called amphisomes, and lysosomes, forming autolysosomes, where finally the cargo is degraded [61,62]. A general phenotype of a defective autophagy is the accumulation of microtubule-associated protein 1 light chain 3B (LC3B) and p62/SQSTM1 (p62), two proteins widely used as autophagy marker proteins. LC3BI and LC3BII, two isoforms of LC3B, are key players in autophagy. During the induction of autophagy, the cytosolic LC3BI is processed into the membrane-bound LC3BII and incorporated into the membrane of autophagosomes, where it is detectable throughout the whole process [63]. Thus, an increased LC3BII/LC3BI ratio hints towards an overall hampered autophagy [36]. Additionally, measurements of p62 level can be used to assess alterations of the autophagic flux. It binds LC3BII and the cargo and is degraded in the autolysosome. Therefore, an accumulation is used as a sign for impairments of the autolysosomal activity [36]. Alterations in lipid composition are likely to affect the autophagic pathway as autophagy is involved in lipid metabolism [64] and might be of special interest regarding neurodegenerative diseases. Autophagy is reported to be implicated in the pathogenesis of different neurodegenerative diseases, such as Alzheimer´s disease (AD) [65], Parkinson´s disease (PD) [66], Huntington´s disease [67] and amyotrophic lateral sclerosis (ALS) [68].

Regarding NPC1, alterations in the autophagic pathway were first reported in NPC1-deficient mice [69,70] and NPC1 patient-derived fibroblasts [71,72], and later in pluripotent stem cell-based model systems. In 2012, Ordonez and colleagues [32] suggested an impaired autophagy in human embryonic stem cell(hES)-derived NPC1-deficient neurons. Serum deprivation of NPC1-deficient neurons and fibroblasts triggered a severe increase in the LC3BII/LC3BI ratio, which was slightly increased under treatment with the lysosomal inhibitor leupeptine, pointing at a decreased degradation rate of autophagosomes. The authors proposed a model combining the induction of autophagy and inhibited clearance of autophagic vesicles. Treatment with the cholesterol mobilizing drug Methyl-β-cyclodextrin (M-β-CD) reduced the LC3BII/LC3BI ratio, linking the cholesterol accumulation, which is typically seen in NPC1, to defects in autophagy. In 2014, Maetzel and colleagues [33] investigated autophagy in iPSC-derived neurons and hepatocyte-like cells. The authors described increased levels of p62 and LC3BII under basal conditions in both cell types, and electron microscopy revealed an increased amount of autophagic vacuoles. Cells with a TALEN-mediated correction of the NPC1 mutation were indistinguishable from control cells. After treatment with bafilomycin A1, an inhibitor of the vacuolar-type H^+^-ATPase, to block the clearance of autophagic vesicles, the LC3BII/LC3BI ratio was similar between NPC1-deficient and control cells. Thus, an increased autophagosome production, causing an accumulation of autophagic vacuoles, is unlikely. The authors suggest that a hampered cholesterol metabolism affects the autophagic flux, which in turn increases the accumulation of lipids, trapping the cells in a vicious cycle. The cells were rescued by the induction of autophagy or cholesterol depletion, restoring proper lipid metabolism. Different autophagy inducers, such as rapamycin (Rap), carbamazepine (CBZ), verapamil (Ver), trehalose (Tre) and SMER28, were tested as potential drugs. In hepatocyte-like cells, only CBZ showed a positive effect similar to Rap on the p62 level and the number of apoptotic cells. Interestingly, in neuronal cells all tested potential drugs could rescue the two mentioned phenotypes, indicating a cell type specific impact of the compounds.

Lee and colleagues [35] investigated the effect of an abnormal vascular endothelial growth factor (VEGF)/Sphingosine kinase (SphK) pathway and sphingosine levels on autophagy in human iPS-derived NPC1-deficient neurons. They found reduced VEGF levels and consequently reduced SphK activity, leading to sphingosine accumulation and reduced sphingosine-1-phosphate (S1P) levels. Treatment with recombinant VEGF led to a correction of these phenotypes and to a decreased level of unesterified cholesterol. The authors suggested that VEGF-mediated sphingolipid changes affect autophagic activity. Indeed, they found increased levels of autophagic markers LC3BII and p62. Treatment with recombinant VEGF decreased the protein level of these markers and decreased autophagosome accumulation, suggesting that VEGF elevates autophagosome-lysosome fusion. Thus, VEGF demonstrates a potential therapeutic intervention strategy for NPC1 disease.

Elevated LC3BII and p62 levels were also described by Soga and colleagues [36]. The authors analyzed neurons and hepatocyte-like cells derived from patient-specific iPSCs. Treatment with hydroxypropyl-β-cyclodextrin (HP-β-CD) and hydroxypropyl-γ-cyclodextrin (HP-γ-CD) resulted in a correction of the cholesterol accumulation, as well as LC3BII and p62 levels. This could be explained by the fact that the restoration of normal cholesterol content in the cell could affect the membrane composition of autophagosomes and lysosomes, leading to normalization of autophagic flux, and thus normalization of LC3BII and p62 levels. However, the exact mode of action of cyclodextrins (CDs) is not yet completely understood. Interestingly, the FDA approved drug miglustat showed no effect.

Dai and colleagues [42] used fibroblasts, neural stem cells and iPSC-derived neurons to analyze the effect of methyl-β-cyclodextrin (M-β-CD) on autophagy and its underlying mechanism of action. In all three cell types, basal levels of LC3BII and p62 were increased. Bafilomycin A1 treatment led to a further increase in control cells and NPC1-deficient cells, whereas M-β-CD only elevated the levels in NPC1-deficient cells, pointing at an impaired fusion of autophagosomes and lysosomes. Time course studies with M-β-CD also revealed a time dependent decline in cholesterol, correlating with changes in the LC3BII and p62 levels. One can speculate that M-β-CD initially induces autophagosome formation and later rescues their clearance by the mediation of cholesterol reduction. Evaluation of the mechanism of M-β-CD showed an activation of AMP-activated protein kinase (AMPK) by direct binding to PRKAB1 or PRKAB2 (encoding the AMPK β1 or β2 subunit), resulting in an inhibition of mechanistic target of rapamycin complex 1 (MTORC1) and an induction of autophagy [42].

Autophagy appears to be a central element in the pathogenesis of NPC1 and seems to be linked to many of the pathophysiological signs observed. All studies about autophagy in NPC1 depicted the accumulation of LC3BII and p62 as a result of an inhibited clearance of autophagosomes due to an inhibited fusion of autophagosomes and lysosomes, displaying a major component of the overall defective autophagy. As the lipid composition of organelles is important to ensure their proper function, the role of cholesterol in autophagy is another key aspect and might connect the reduced NPC1 activity to autophagy [33]. In other diseases such as ALS [73], AD [74] and PD [75], an induction of autophagy is discussed to support the survival of affected cells. Although treatment strategies aiming at targets downstream of MTORC1 appear to be promising, they might be harmful as many other cellular pathways can be affected, leading to severe adverse effects [76]. In addition to defects in autophagy, an accumulation of some kind of storage material is a pathophysiological phenotype observed in many neurodegenerative diseases. Improving the clearance of such material by activating the autophagic pathway could represent a general treatment strategy, and this underlines the importance of autophagy in current research on neurodegenerative diseases.

### 3.3. Defective Mitochondrial Homeostasis and Oxidative Stress

Mitochondrial function and oxidative stress (OS) are closely linked, as mitochondria are not only susceptible to OS, but can in return elevate the level of reactive oxygen species once their function is hampered. Not surprisingly, mitochondrial function and OS are hallmarks of neurodegenerative diseases such as Alzheimer´s disease and Parkinson´s disease [77], as well as NPC1 [78]. Regarding mitochondrial function and oxidative stress in NPC1, a series of publications deal with these topics. Although currently only one publication, by Jürs and colleagues [45], has analyzed OS in iPSC-derived neuronal cells, we will briefly summarize the results obtained with other NPC1 model systems, as the restoration of mitochondrial function and the improvement of OS are discussed as possible targets for the treatment of neurodegenerative diseases (for review see, e.g., [79,80]).

Ordonez and colleagues [81] suggested that an increase in autophagy with a simultaneous disturbance of the autophagic flux is the cause of mitochondrial fragmentation observed in NPC1-deficient neurons derived from NPC1 knock-down human embryonic stem cells. They found an accumulation of partially degraded, metabolically inactive mitochondrial fragments. This fragmentation was shown to be downstream of autophagy as the phenotype could be rescued by the inhibition of autophagy with 3-methyladenine (3-MA). Yu and colleagues [82] described an elevated level of cholesterol in the mitochondrial membrane of neurons in NPC1-deficient mice. They concluded that the membrane integrity and membrane potential are hampered by the elevated cholesterol level, causing a general reduced mitochondrial function, resulting in a decreased ATP synthase activity and therefore a reduced ATP production. Treatment of the NPC1-deficient mice with methyl-β-cyclodextrin restored the ATP synthase activity by lowering the cholesterol level in the mitochondrial membrane [82]. Marí and colleagues [83] evaluated the mitochondrial phenotype of hepatocytes of NPC1-deficient mice and revealed an 8- to 10-fold higher level of free cholesterol. In addition, increased cholesterol levels were observed in mitochondria, suggesting that this could be the cause of reduced mitochondrial glutathione and impaired antioxidant capacity. Interestingly, the endoplasmic reticulum (ER) shows a reduced cholesterol content, which indicates an incorrect transport of free cholesterol to the ER. This could cause ER stress and ultimately trigger the unfolded protein response [83]. As alterations of the mitochondrial cholesterol level can lead to mitochondrial dysfunction and alterations of the mitochondrial respiratory chain, an occurrence of reactive oxygen species and oxidative stress appears to be obvious [84]. In regards to oxidative stress, Fernández and colleagues [85] described an increased susceptibility of mitochondria in NPC1-deficient mice, as treatment with amyloid β1-42 peptide resulted in elevated levels of reactive oxygen species (ROS). Additionally, an elevation of cytochrome c and Smac/DIABLO levels, in the supernatant of isolated mitochondria, hints towards the induction of apoptosis [85].

There have been several studies investigating the role of oxidative stress in fibroblasts derived from NPC1 patients. Zampieri and colleagues [86] described an elevated ROS level in NPC1 patient-derived fibroblasts, induced by cholesterol accumulation, which could be mimicked by siRNA mediated knockdown of NPC1. The activity of catalase, a major component of the cellular anti-oxidative response, was decreased, and an elevated lipid peroxidation was observed. Moreover, NPC1-deficient fibroblasts appeared to be more susceptible to apoptosis, induced by treatment with H_2_O_2_. Allopregnanolone rescued the fibroblasts from the described phenotype linked with oxidative stress, but had no effect on the cholesterol and glycosphingolipid levels, suggesting pleiotropic effects of allopregnanolone [86].

Fu and colleagues [78] analyzed serum from a cohort of NPC patients and investigated oxidative stress by determining coenzyme Q10 (CoQ10) and Trolox Equivalent Antioxidant Capacity (TEAC). The reduced form of CoQ10 is a coenzyme of the mitochondrial protein complexes I, II and III, which are involved in the antioxidant defense system, while the TEAC describes the antioxidant capacity of the cell. A lowered amount of reduced CoQ10 and altered TEAC was found in the serum of the NPC patients, but this could not be influenced by CoQ10 supplementation or miglustat treatment [78].

Cologna and colleagues [87] analyzed the expression of proteins in the cerebellum of NPC1-deficient mice. Amongst 77 proteins found to be differentially expressed in comparison to control mice were proteins known to be involved in the glutathione metabolism and the anti-oxidative defense system, such as glutathione S-transferase (GST), glutathione S-transferase alpha 4, glutathione S transferase pi1 and mitochondrial isocitrate dehydrogenase 2 (NADP^+^). The cerebrospinal fluid of NPC1 patients was examined regarding the expression of glutathione S-transferase alpha 4 and superoxide dismutase I (SOD1). GST alpha 4 levels were reduced, while the levels of SOD1 were increased, indicating that cells of the central nervous system (CNS) of NPC1 patients suffer from oxidative stress [87]. 

Regarding studies investigating oxidative stress in iPSC-based model systems, currently only one study is published. Recently, Jürs and colleagues [45] analyzed oxidative stress in an iPSC-based neuronal model system. Here, we detected an elevated ROS level in NPC1-deficient cells harboring different NPC1 mutations, amongst them the mutation I1061T. A hampered efficacy of the anti-oxidative defense system was further shown by an elevated nitration of proteins and reduced superoxide dismutase (SOD) activity. In addition, a reduction in SOD1 and SOD2 levels was described, as well as almost a complete lack of catalase in the NPC1-deficient cells. The authors conclude that this striking lack of catalase could be the cause of oxidative stress. Although these results, consistent with results from mouse NPC1 models or fibroblasts derived from NPC1 patients, demonstrate the value of iPSC-based model systems, further studies are needed to clarify the influence of oxidative stress on neurodegeneration in NPC1.

### 3.4. Gliosis

Gliosis is a physiological response in the central nervous system as a result of tissue damage. Initially, gliosis has been shown in post-mortem studies [14,15] and mice models [21,88] for NPC1, describing an increased number of reactive astrocytes. According to these findings, an iPSC-based neuronal cell system confirmed changes in glial activation. Peter and colleagues [41] used an iPSC-based neuronal cell system to investigate gliosis in different homozygous and compound heterozygous NPC1 mutated cell lines. We observed higher amounts of GFAP^+^/vimentin^+^ cells, as well as an increased protein amount of both intermediate filaments. In contrast, cells obtained via the direct conversion of fibroblasts into neural stem cells [49] showed no differences in the expression of GFAP, but there were no further studies on gliosis in this work. Peter and colleagues [41] described cells double positive for GFAP and Ki67 reflecting proliferative reactive astrocytes. In addition, GFAP and vimentin had been shown to be hypophosphorylated, subsequently leading to a polymerization of intermediate filament monomers, resulting in a criss-cross-like appearance of vimentin and GFAP in the cytosol. Regarding vimentin, comparable results were described for NPC1 patient-derived fibroblasts [89,90]. The authors described alterations of vimentin based on its hypophosphorylation due to a decreased activity of protein kinases. Treatment of the NPC1-deficient fibroblasts with Phorbol 12-myristate 13-acetate (PMA), an activator of protein kinase c, ameliorated the hypophosphorylation of vimentin as well as the hampered assembly of the intermediate filament. As shown by Peter and colleagues [41], treatment with PMA resulted not only in a correction of the observed hypophosphorylation of vimentin and GFAP, but it also rescued the iPSC-based neuronal cell system from gliosis and cholesterol accumulation. The results of these studies suggest that compounds activating protein kinases display an interesting treatment strategy and, consequently, such compounds are claimed for the treatment of lysosomal storage disorders [91].

### 3.5. Functional Aspects of NPC1-Deficient Neurons

NPC1 patients suffer from various neurological symptoms, such as ataxia [1]. The ataxia observed in NPC1 patients is based on the selective death of cerebellar Purkinje cells (cPCs). Investigations on the neurodegenerative processes underlying this cell death were performed in NPC1-deficient mice, with the mouse strain Npc1^nih^ (BALB/cNctr-NPC1m1N/J) being the most commonly used [12]. In addition to a reduced lifespan [92,93], these mice are mainly characterized by pronounced ataxia based on selective Purkinje cell death, which starts anteriorly in lobe 1 of the cerebellum and spreads posteriorly to lobe 9. Lobe 10 does not appear to be affected by neurodegenerative processes [94]. A similar selective death of cPCs can be observed in the group of hereditary spinocerebellar ataxias [95]. These inherited diseases are characterized by a progressive loss of cPCs, the causes being various genetic defects, such as mutations in voltage-activated potassium channels. Such mutations ultimately lead to changes in cPCs at the functional level. cPCs are characterized by the ability to generate intrinsic action potentials. The formation of these firing patterns is independent of synaptic input, but it is modulated by them. Changes in the firing patterns have been described for spinocerebellar ataxias, among others, and probably contribute to the destruction of the cPCs. To what extent changes in the intrinsically generated firing patterns of cPCs exist and to what extent they contribute to the pathophysiological mechanisms of NPC1 are poorly understood. We identified changes in the intrinsically generated firing patterns in NPC1-deficient adult mice affected by ataxia [19] and changes in the synaptic modulation of cPCs prior to the onset of ataxia [20,96]. Inhibitory GABA_A_ receptors and excitatory AMPA-receptors appear to be involved. We conclude that changes in synaptic modulation may contribute to or trigger cell loss, but the exact mechanism remains to be elucidated.

Regarding the use of iPSC-derived neurons, we have undertaken a functional study on NPC1-deficient neurons [20]. First of all, this study proved that NPC1 patient-specific iPSCs can be differentiated into functional neurons, which was shown by the expression of voltage-activated and ligand-activated ion channels. Furthermore, the cells are able to form neural networks. Interestingly, the authors found differences in AMPA-receptor (AMPAR) mediated currents. Both the current responses and the AMPA-induced increases in the intracellular Ca^2+^-concentration were lower in NPC1-deficient cells. This was probably based on a higher amount of Ca^2+^-impermeable AMPARs, containing the GluR2-subunit. The authors were able to show that NPC1-deficient cells had an increased proportion of membrane-bound GluA2 subunits and, as a consequence, the Ca^2+^ permeability of AMPARs was reduced. An altered stoichiometry of the subunit composition of AMPARs was also described in the cell cultures of primary neurons of NPC1-deficient mice [97]. These neurons also showed changes in their neuronal network activity [98], which supports the hypothesis that such changes may well contribute to the neurodegenerative processes of NPC1.

This work on iPSC-derived NPC1-deficient neurons, but also studies on iPSC-derived neurons in the context of other neurodegenerative diseases, shows that iPSCs are a valuable source for studies on human cell types that are otherwise not available or can only be obtained infrequently and/or with great difficulty. It is expected that future work with such iPSC-based models will contribute significantly to the elucidation of pathophysiological processes, not only in NPC1.

## 4. Treatment Strategies for NPC1 and the Use of iPSCs for Drug Discovery

Pluripotent stem cells are undoubtedly a powerful tool for disease modeling and drug discovery because they provide unlimited access to affected cells that are difficult to obtain from patients, such as those of the nervous system. This holds especially true for disease modeling and drug discovery in the field of rare diseases, such as lysosomal storage disorders. However, the limited availability of patient-derived biopsies is pushing this method to its limits. With regard to NPC1, this is probably reflected in the fact that the number of studies using iPSCs for drug testing is currently low. As a side note, it is probably not the lack of patient samples that hinders rare disease research, but the lack of coordination of sample collection making these samples available for rare disease research worldwide. However, there are now international initiatives to provide iPSC lines, such as the European Bank for induced pluripotent Stem Cells (https://ebisc.org/), that could help to overcome these obstacles. Nonetheless, we provide below an overview of the strategies currently under discussion as effective therapies to ameliorate the pathophysiological manifestations of NPC1. We summarize not only studies that involve the use of patient-derived iPSCs, but also studies that use other NPC1 models, such as patients’ fibroblasts or murine models. We focus on the use of miglustat, cyclodextrins, histone deacetylase inhibitors, and pharmacological chaperones as potential therapeutic approaches for NPC1.

### 4.1. Administration of Miglustat

To date, miglustat (*N*-butyl-deoxynojirimycin; Zavesca®), originally developed as a treatment for Gaucher’s disease [99], is the only drug available for the treatment of NPC1 that has been approved by the European Union [100]. Although miglustat has not yet been approved by the FDA, it is also used for the off-label treatment of NPC1 in the United States. Miglustat was designed as a substrate reduction therapy aiming to reduce the synthesis of material stored in Gaucher’s disease. Miglustat is an iminosugar acting as an inhibitor of the glycosylceramide synthase, which plays a major role in the synthesis of a large number of glycosphingolipids [101].

In NPC1-deficient mice, miglustat showed promising effects on the NPC1 phenotype, namely slowing down neurological decline and prolonging the survival time of the animals [102]. An open-label, randomized trial that aimed to evaluate the efficacy, safety and tolerability of miglustat (International Standard Randomized Controlled Trial ISRCTN26761144) [103] was initiated as a phase I/II clinical trial. The study also included an additional pediatric cohort of children <12 years. The primary endpoint was horizontal saccadic eye movement (HSEM) velocity. At 12 months, the HSEM had improved in the miglustat group compared to the control group. Furthermore, an improvement in swallowing capacity, stable acuity of hearing and a slower deterioration in ambulatory index was observed [103]. These effects could be confirmed in two extension studies [104,105]. The most frequent adverse events observed by miglustat are mild gastrointestinal effects, such as diarrhea, flatulence and abdominal pain as well as weight loss, and tremors [101,106]. Regarding NPC1-deficient iPSCs, two studies analyzed the effect of miglustat. Yu and colleagues [34] did not observe any beneficial effect after the treatment of iPSC-derived neurons. The treatment with 30 µM miglustat had no effect on cholesterol accumulation, analyzed by filipin staining. Soga and colleagues [36] compared the influence of miglustat and cyclodextrins on cholesterol accumulation, altered autophagy and altered ATP levels in neural precursor cells and HLCs. The authors also could not observe an influence of miglustat. Treatment with 50 µM or 500 µM miglustat for 4 days did not impact any of the parameters analyzed, although they confirmed an inhibition of glycosylceramide synthesis. In contrast, HP-β-CD and HP-γ-CD showed a positive effect on the investigated pathophysiological parameters, indicating a different mechanism of action of cyclodextrins. We can only speculate about the discrepancy between the results of the clinical studies and the studies on iPSCs. The fact that the inhibition of glycosylceramide synthesis was shown, but no positive effect was observed on iPSC-derived cells, may be evidence that these model systems represent the pathophysiology of NPC1 only to a certain extent. Even too low doses chosen for the in vitro studies or insufficient application times could have an impact on the differences between the clinical studies and the studies with iPSCs. Moreover, effects such as the first pass effect or the distribution of drugs in the body cannot be reproduced in such models.

Despite the sobering results obtained with miglustat in human NPC1-deficient iPSC-derived neurons, one should consider the promising results showing the stabilization of neurological manifestations in pediatric and adult NPC1 patients. Thus, miglustat represents the most valuable agent currently available for the treatment of NPC1 and, in a sense, defines the standard that should be surpassed by new therapeutic approaches.

### 4.2. Cyclodextrins

Cyclodextrins (CDs) are cyclic oligosaccharides that are made up of (α-1,4)-linked glucopyranose units [107]. The most common CDs are α,β and γ-CD which consist of six, seven and eight glucose units, respectively [108]. CDs have a hydrophilic outer surface and a hydrophobic inner cavity [109], and thus are very well suited to increase the bioavailability of poorly soluble substances [110]. 

Regarding NPC1, HP-β-CD was used to solubilize allopregnalone in a study analyzing the impact of allopregnalone on NPC1-deficient mice [111]. Treatment of NPC1-deficient mice resulted in a significantly prolonged lifespan and a slowdown of the progressive neurodegeneration [111]. Interestingly, a single treatment of NPC1-deficient mice with HP-β-CD prolonged the life span of the mice, but additional treatment with allopregnanolone had no beneficial effect [112]. These results suggested a positive effect of the solvent HP-β-CD itself. Subsequent studies showed that HP-β-CD alone was more efficient than the suspected drug allopregnalone [93,112,113] in terms of reduced cholesterol and sphingolipid storage, extended lifespan, improved liver function, and neurodegeneration. However, an intrathecal administration of CDs appears to be a prerequisite for successful treatment, as CDs are not able to efficiently cross the blood-brain barrier [114].

Regarding the use of iPSC-derived cells, CDs have been shown to modulate defective autophagy, observed in NPC1, but the mechanisms underlying the effects of CDs on autophagy are discussed differently. Maetzel and colleagues [33] demonstrated a concentration dependent effect of HP-β-CD. While low concentrations (0.2%) were sufficient to induce cholesterol esterification, they did not interfere with autophagy in iPSC-derived HLCs. Higher doses (1%) did not correct the disturbance in the autophagic flux but blocked autophagy, as demonstrated by the increase in LC3BII and p62 levels. The combination of low doses of HP-β-CD and carbamazepine rescued both autophagy and cholesterol accumulation in iPSC-derived HLCs and neurons [33], thus a combination of cholesterol reduction by CDs and autophagy stimulation by CBZ might be a promising treatment strategy for NPC1. Dai and colleagues [42] affirmed a positive effect of M-β-CD on autophagy. They demonstrated that M-β-CD activates the β-subunit of the AMP-activated protein kinase (AMPK), and thereby activates the autophagic flux and reduces cholesterol content. Further studies also described an effect of CDs in human iPSC-based neuronal and hepatic cell systems. Ordonez and colleagues [32] observed a reduced LC3BII/I ratio by Western blot analysis after the treatment with HP-β-CD, suggesting that the mobilization of cholesterol from lysosomes increases the turnover of LC3B. In addition, they observed reduced p62 punctae and mitochondrial fragmentation. Cyclodextrins have also been part of the study of Yu and colleagues [115], using a transgene-free reprogramming method based on Sendai virus. One control and one compound heterozygous fibroblast cell line were reprogrammed to iPSCs, differentiated into neural stem cells and neurons, and subsequently used for drug screening. Among nine agents tested, they found HP-β-CD, M-β-CD and δ-tocopherol to effectively reduce cholesterol accumulation. This was shown by reduced filipin staining and reduced lysotracker staining, indicating a reduction in enlarged lysosomes. Of note, HP-β-CD was more potent in neurons compared to fibroblasts, although they were derived from the same patient. These results indicate not only cell type specific differences, but the necessity of using disease-affected cell types, such as neurons and hepatocyte-like cells in case of NPC1, for disease modeling and drug discovery.

The most recent study regarding cyclodextrins was performed by Soga and colleagues [36]. The authors tested the three most common CDs: α, β and γ. While HP-α-CD had no effect, HP-β-CD and HP-γ-CD reduced cholesterol in iPSC-derived HLCs to the same extent and the size of the enlarged NPC1-deficient HLCs was decreased. The differential effect of these CDs might be explained by the different cavity size of the CDs. While the hydrophobic inner space of HP-α-CD is comparatively small, the cavity of γ and β-CD might better incorporate the side chain of cholesterol. While low concentrations (100 µM) were inefficient, higher concentrations (1 mM) did not only reduce the cholesterol content, but also recovered lowered ATP levels, and a positive impact on autophagy was shown by a reduction in elevated LC3BII and p62 levels in HLCs and neural progenitors. HP-γ-CD could rescue the abnormal expression pattern of genes closer to the normal pattern than HP-β-CD, and therefore appeared to be more effective. 

The exact mode of action of CDs is not yet understood, but several mechanisms are discussed, such as a drain of cholesterol incorporated in cell membranes that subsequently needs to be replaced by intracellular cholesterol, thus decreasing cholesterol accumulation. Rosenbaum and colleagues [115] discussed the endocytosis of CDs, specifically by pinocytosis, and subsequently complexing cholesterol.

The promising results of studies using animal models, as well as iPSC-based models, initiated clinical trials using two derivates of HP-β-CD, namely VTS-270 (Vtesse, Inc., Gaithersburg, MD, USA) and Trappsol® Cyclo™ (CTD Holdings, Inc., Alachua, FL, USA), receiving orphan drug designation by the FDA and EMA. The active ingredient of VTS-270 is Kleptose® HPB (Roquette Pharma, Lille, France). The first phase I/IIa clinical trial was started in 2013 (https://clinicaltrials.gov/ct2/show/NCT01747135). This non-randomized, open-label, dose-escalation phase I/IIa study aimed to evaluate the safety and clinical efficacy of the intrathecally administered HP-β-CD derivative VTS-270 [116]. Doses ranging from 50–1200 mg were evaluated in 14 NPC1 patients with neurological involvement (age 4–23 years). Patients were treated monthly for 12–18 months. To explore the potential of a biweekly treatment strategy, three additional participants were treated with HP-β-CD. Dose was initiated at 50, 200, 300 or 400 mg HP-β-CD in groups of three participants, and two were initially dosed at 900 mg. Doses were subsequently escalated on the basis of tolerance to 600 or 1200 mg. As the primary outcome measure, 24(S)-hydroxycholesterol (24(S)-HC) concentration was measured in plasma and cerebrospinal fluid. The 24(S)-HC served as a biomarker for improved cholesterol homeostasis in neurons, as it is derived almost exclusively from neurons in the CNS. The NPC1 Neurological Severity Scores (NSS) were used to compare disease progression relative to a comparison cohort of 21 NPC1 participants of similar age range. All major NSS domains showed decreased or stabilized NSS in 10/17 participants. A decrease in neurological disease progression was observed in NSS ambulation, cognition and speech subdomains. No serious adverse events were observed. As an expected adverse event, mid-to high-frequency hearing loss was documented in 100% of the participants, likely due to outer hair cell loss. This was managed with hearing aids. Taken together, this clinical phase I/IIa trial demonstrated an acceptable safety profile and slowing of disease progression [116]. Furthermore, cognitive and adaptive skills did not significantly decline during the trial [117]. In July 2015, a phase II/III prospective, randomized, double-blind, Sham-controlled clinical trial (NCT02534844; https://clinicaltrials.gov/ct2/show/NCT02534844) was conducted to evaluate the efficacy and safety of HP-β-CD, administered intrathecally, in patients with neurological manifestation. 

A further phase I/II clinical trial with an intravenous administration of VTS-270 was approved in 2018, dealing with infantile liver disease (https://clinicaltrials.gov/ct2/show/NCT03471143). Two clinical trials administered Trappsol® Cyclo™, a derivative of HP-β-CD, intravenously: a phase I clinical trial that involved 12 adults ≥ 18 years (https://clinicaltrials.gov/ct2/show/NCT02939547) and a phase I/II clinical trial, currently recruiting participants ≥ 2 years (https://clinicaltrials.gov/ct2/show/NCT02912793). 

In summary, the studies with iPSCs have shown that cyclodextrins are a promising treatment approach for NPC1. However, the results of the clinical trials must be put into perspective by studies with NPC1 models, as harmful side effects must be taken into account, such as ototoxicity, which has been observed in murine NPC1 models [118]. Nevertheless, the studies of CDs with iPSC-based models show their suitability for drug discovery.

### 4.3. Histone Deacetylase Inhibitors 

The balance of histone acetyl transferases (HATs) and histone deacetylases (HDACs) defines the acetylation status of histones and thereby plays a key role in gene regulation. HATs and HDACs perform reversible post-translational modifications. While HATs cause chromatin relaxation, making DNA promoter regions more accessible for transcription activators, HDACs cause a compact DNA formation, and subsequently a repression of genes [119]. An imbalance between histone acetylation and deacetylation has been associated with cancer [120] and neurodegenerative diseases [121].

To date, four histone deacetylase inhibitors (HDACis) are approved by the FDA for different diseases, including suberoylanilide hydroxamic acid (SAHA; also known as Vorinostat) and FK-228 (Romidepsin) for the treatment of cutaneous T-cell lymphoma (CTCL), LBH589 (Panobinostat) for the treatment of CTCL and multiple myeloma, and PXD101 (Belinostat) for the treatment of peripheral T-cell lymphoma (PTCL).

Regarding NPC1, several studies showed the advantageous effect of HDACis on the NPC1 phenotype in different cell models. Since most HDACis are able to cross the blood-brain barrier, they represent a promising strategy with unproblematic administration for the treatment of the neurological manifestations of NPC1. The treatment of NPC1-deficient mice with the HDACi valproic acid [122] improved the cholesterol homeostasis. Munkacsi and colleagues [123] identified upregulated HDAC genes in NPC1 patient-derived fibroblasts. The HDACi SAHA was able to reverse the dysregulation of these genes and decrease lysosomal cholesterol accumulation. Pipalia and colleagues [124] evaluated the effect of six different HDACis in NPC1 patient derived fibroblasts. SAHA and LBH598 were not only sufficient to reduce cholesterol accumulation, but also to restore cholesterol homeostasis, shown by the normal processing of sterol regulatory-binding protein 2 (SREBP2) and a reduction in LDL receptor expression. The authors did not observe an effect in NPC2 mutated cell lines, thus concluding that HDACis do not overtake the function of NPC1 or NPC2 proteins. However, the mechanisms of action of HDACis are poorly understood. Interestingly, the sphingolipid metabolite sphingosine-1-phosphate (S1P) is an endogenous inhibitor of class I HDAC1 and HDAC2 [125]. The authors suggested that because of the accumulation of sphingosine in lysosomes, less sphingosine is available for phosphorylation to S1P, which leads to enhanced activity of HDAC1 and HDAC2. In 2014, a phase I/II clinical trial was approved to determine the safety and tolerability of vorinostat, as well as to determine the biochemical efficacy to increase the expression of NPC1 protein and normalize lipid and protein biomarkers (https://clinicaltrials.gov/ct2/show/NCT02124083). In this non-randomized, open-label study, 12 participants were involved to test orally administered vorinostat in a 3 days on/4 days off schedule. For the first three months, participants were dosed with 200 mg followed by dose escalation to 400 mg for another 3 months.

Using an iPSC-based model, until now, HDACis have been investigated only by Yu and colleagues [34]. Despite the promising results from fibroblasts and animal models, no effect on the cholesterol level could be observed on the iPSC-derived neuronal cells after the treatment with 1 µM SAHA, as shown by an unchanged filipin staining.

Similar to the studies on CDs, the limited number of studies on iPSCs with regard to the use of HDACis is currently insufficient to make a final decision on the usefulness of iPSC-based models. However, as iPSCs are successfully used for disease modeling in NPC1, one must be optimistic that future studies will show the usefulness of iPSC-based models in connection with the use of HDACis as NPC1 therapy.

### 4.4. Pharmacological Chaperones

To anticipate the bad news: there are no studies that prove the effectiveness of pharmacological chaperones (PCs) in cell models based on iPSCs. However, we would like to summarize the data on the efficacy of PCs, obtained with other NPC1 model systems, as they are very promising and support the use of PCs as a treatment strategy for NPC1.

PCs are small molecules and therefore have the advantageous property of crossing the blood-brain barrier, making their use in the treatment of neurodegenerative diseases conceivable [126]. In addition, they show high bioavailability and their high specificity allows their use in low concentrations compared to more general functional chemical chaperones [127]. They can be administered orally and therefore have little, if any, impact on the quality of life of patients. Pharmacological chaperones bind to folding intermediates of proteins processed in the endoplasmic reticulum, stabilizing them and thus protecting them from degradation, ultimately enabling correct intracellular localization. With regard to mutated proteins, they can lead to an increase in the cellular active pool of the actually mutated but still active protein. While several compounds are discussed to work as PCs in several lysosomal storage disorders (LSDs) (for review, refer to [127]), only a few studies are available for NPC1, although the use of PCs for the treatment of NPC1 appears to be very attractive, as a significant proportion of NPC1 mutations lead to misfolded proteins. Such misfolded NPC1 proteins are recognized by the quality control system of the endoplasmic reticulum and then marked for proteasomal degradation. However, these mutated NPC1 proteins may have a cholesterol transport activity, albeit reduced. The prevalent mutation I1061T, found in a European cohort [128], fulfils the criteria of a misfolded but still active protein, which was demonstrated by Gelsthorpe and colleagues [129]. The authors showed that the mutated protein I1061T exhibits reduced stability and thus reduced activity, but not a complete loss of function. Therefore, the NPC1 mutation I1061T seems to be the best choice to test the concept of using pharmacological chaperones as a treatment strategy for NPC1.

Ohgane and colleagues [130] identified different oxysterols, such as 25-hydroxycholesterol (25-HC), and other derivatives to act as pharmacological chaperones. Treatment with 25-HC resulted in the correction of maturation and localization of the NPC1 protein and reduced cholesterol accumulation in NPC1-deficient patient fibroblasts harboring the I1061T mutation. The authors showed that these oxysterols bind to a site that is different from the N-terminal sterol binding site, implicating a second sterol-binding site. In a further study, the authors aimed to identify other non-steroidal pharmacological chaperones, expecting a better metabolic stability and a higher selectivity towards NPC1 over other sterol-binding proteins, and identified non-oxysterols as pharmacological chaperones for the treatment of NPC1 [131].

Encouraged by these findings, existing compound libraries could be screened for potential pharmacological chaperones. The ideal chaperone for the treatment of NPC1 has a high affinity to the misfolded protein in the ER to assist folding and, subsequently, shuttling to the LE/LY, but also displays good dissociation properties from the protein in the acidic environment of lysosome to prevent inhibition of the cholesterol binding pocket, enabling a rescue of NPC1 transport function. These prerequisites may be tested by in silico binding studies, to reduce the number of compounds to be tested in the wet lab. 

## 5. Limitations and Perspectives of iPSCs Used for Disease Modeling and Drug Discovery

The authors of this review are enthusiastic about the approach of using iPSC-based cell models for disease modeling and drug discovery, especially for the neurovisceral disease NPC1. However, we are also aware of the limitations of using iPSC-based model systems and therefore want to address at least some of these issues.

### 5.1. Limitation of the Applicability of iPSCs and Derived Cells 

Since the ground-breaking publications by Yamanaka and colleagues in 2006, describing the reprogramming of mouse fibroblasts into induced pluripotent stem cells [132], and of human fibroblasts in 2007 [133,134], a range of methods combining the use of different donor somatic cells, transcription factors, gene delivery methods and small molecules have been developed to generate disease specific iPSC lines. To date, the most popular donor somatic cells are fibroblasts, being used in more than 80% of all reprogramming experiments published [135]. Additionally, other cell types have been used for reprogramming, such as human primary keratinocytes, peripheral blood mononuclear cells, and urine-derived renal epithelial cells [136,137,138]. In general, somatic cells are discussed to have an “epigenetic fingerprint”, reflected by a pattern of chemical markers on their DNA, impacting the reprogramming and, later, the differentiation potential of the cells. Furthermore, iPSCs represent cells in an embryonic developmental stage, and, thus, one must keep in mind some drawbacks related, e.g., to the maturation of iPSC-derived neurons. This is of particular interest for neurons because the expression of ion channel subunits changes during embryonic/fetal development. For example, the subunit composition of inhibitory GABA_A_ receptors (GABAAR) and glycine receptors (GlyR) changes during the maturation of neurons, and thus the functional properties, such as the activation/inactivation and ion conductance of the ion channels, are altered. Moreover, GABAAR and GlyR are excitatory rather than inhibitory at early developmental stages. This is due to increased intracellular Cl^−^-concentration achieved by Cl^−^-transporters expressed during embryonic development [139]. Our group observed GABA-induced depolarization in the iPSC-derived neurons used by Rabenstein and colleagues [20], which led to the activation of voltage-gated Ca^2+^ channels (unpublished data), indicating a fetal developmental stage of the neurons even after six weeks of differentiation. Therefore, whenever possible, functional changes in iPSC-derived neurons should be matched with other model systems, such as primary neuronal cultures, that preferably have a developmental stage relevant to the disease under investigation.

Regarding the developmental stage of iPSCs, several approaches have been made to overcome this issue by using stress inducers and proteins pushing forward the ageing of cells [140]. Trans-differentiation of somatic cells, e.g., fibroblasts, into disease-affected cells, such as neurons, offer the possibility to bypass a state of pluripotency, which is linked to the reset of the age of the cells. This aspect is particularly important for diseases with progressive neurodegeneration. Trans-differentiated cells show no tumorigenicity when transplanted in vivo, but show similar functionality [141,142]. Still, there are limitations in the concept of trans-differentiation, as the obtained population of post mitotic cells, such as neurons, is limited, as such cells do not divide anymore, resulting in a restricted efficiency and expansion for use in high throughput drug screening [142]. 

The choice of the transfection system to obtain iPSCs is without any doubt the most critical step determining the further usability of the iPSCs. While retrovirus was the method initially used for reprogramming, they only infect dividing cells, whereas lentivirus can infect both non-dividing and proliferative cells [143]. The major risk of using retrovirus and lentivirus for reprogramming is the integration of viral vectors into host DNA, and it goes without saying that such iPSCs cannot be used for applications in humans. Non-integrative viral delivery includes adenovirus [144] and Sendai virus [145]. Alternatively, plasmids [146,147], RNA delivery [148] and protein delivery [149] may be used. In addition, several chemical compounds have been shown to further improve reprogramming [150].

After the crucial first step of generating iPSC lines carrying the molecular defects of interest, efficient cell-specific differentiation protocols, in the case of NPC1 with neurovisceral clinical presentation, are required to obtain neuronal and hepatic cells. The use of adequate protocols is challenging, as an almost unmanageable number of differentiation protocols have been published. In terms of NPC1 disease modeling, most neuronal cell systems have been created without specification for differentiation into a particular type of neuron. None of these publications (Table 1) have yet successfully recapitulated, e.g., the specific cerebellar neuronal dysfunction and degeneration. Cerebellar Purkinje cells are particularly susceptible to mutations of the NPC1 protein, and their progressive loss is one of the hallmarks of neurodegeneration observed in NPC1. Due to their complex morphology and circuitry, unique firing properties, and long maturation time, it is certainly challenging to develop appropriate protocols for in vitro differentiation. However, a significant breakthrough has been achieved recently. Muguruma and colleagues [151] described the generation of Purkinje cell precursors from mouse embryonic stem cells, which could be differentiated into neurons showing Purkinje cell-specific characteristics. Silva and colleagues [152] described the maturation of human iPSC-derived cerebellar neurons. The authors developed protocols to generate cerebellar cell types such as Purkinje cells or granular cells, which have been shown to be electrically active cells. It is expected that such model systems will help to decipher the processes underlying the high vulnerability of human Purkinje cerebellar cells, and it is of great interest to what extent human iPSC-derived cerebellar neurons can reflect the in vivo situation. Although model systems based on organoids or 3D-culture systems have great potential to reproduce the in vivo situation in a more complex way than 2D-culture systems, these models also have disadvantages. They can only represent the interaction between different cell types to a certain extent, and the interactions between different organs, affected by a disease, cannot be studied. This is of particular importance, for example, for pharmacokinetics and the distribution of a drug in the organism, especially in neurodegenerative diseases, where potential drugs must be able to cross the blood-brain barrier.

Regarding the differentiation of iPSCs into HLCs, the majority of available protocols are based on three steps: (1) induction of definitive endoderm, (2) specification into hepatic progenitor cells and (3) maturation into HLCs [153,154]. Publications using iPSC-based model systems for NPC1 mostly use one of these strategies, but there are significant differences between the protocols. This has to be considered critically, as it may lead to differences in the appearance of pathophysiological features and their attenuation by potential substances for the treatment of NPC1. 

A further important aspect of using iPSCs is the availability of appropriate control cells. Differences in genetic background between iPSC lines can hamper the interpretation of experimental results. For genetic disorders, genetic editing is a powerful tool to generate gene-corrected iPSCs for disease modeling because it produces cells with identical epigenetic backgrounds. Technologies such as CRISPR/Cas9 can correct or induce a specific mutation, thus leading to an isogenic control that only differs in the underlying mutation compared to its control cell line. In NPC1, it is even more difficult to select an appropriate control, because the pathophysiology of NPC1 is extremely diverse. Selecting mutations to study is particularly difficult in NPC1 because there is no really strong correlation between mutations and phenotypic expression in this disease. Patients with the same mutation, even identical twins affected by NPC1 [155], show strong differences in clinical expression. This example shows that epigenetic aspects can only be poorly represented in cell culture models, and it is questionable whether they play a role at all in iPSC-based models, since the cells undergo an epigenetic reset during reprogramming.

The limitations of iPSCs and their cellular derivatives mentioned above are general and apply to disease modeling and drug discovery for all types of diseases. The wide variety of protocols to generate iPSCs and differentiation into desired cell types make it difficult to compare studies that have used different protocols. On the other hand, iPSC-derived cells used for disease modeling of NPC1 show comparable pathophysiological manifestations or pathogenic mechanisms of NPC1, regardless of the protocols used. Thus, it can be concluded that the observed disease features are due to the NPC1 mutations and not to the protocol used for reprogramming or differentiation, demonstrating the applicability of iPSC-derived cells for disease modeling.

### 5.2. Perspectives of the Applicability of iPSC-Derived Cells

As summarized above, several laboratories showed that iPSC models are a valuable tool for NPC1 disease modeling, revealing pathophysiological features and, moreover, elucidating the pathogenic mechanisms of NPC1. 

Overall, in the studies reviewed here, all major hallmarks of NPC1 have been observed. With one exception, iPSC-based NPC1 disease models showed an increased cholesterol level—the main characteristic feature of NPC1. The same holds true for increased levels of LC3BII and p62, indicating disturbances of autophagy in NPC1. Autophagy is closely related to mitochondrial functions, as the degradation of damaged mitochondria by mitophagy is a major process of the autophagic system in the cell. A proper clearance of damaged mitochondria is essential to maintain cellular homeostasis, especially as different pathophysiological phenotypes, linked to mitochondria, have been observed in NPC1. To cut a long story short, the proof of major pathophysiological features of iPSC-based model systems for NPC1 demonstrates the applicability of such systems. However, the number of studies involving neurons or glial cells is currently low, although these cell types are, besides hepatocytes, the most affected cells in NPC1. 

NPC1, like other neurodegenerative diseases, is characterized by the selective loss of neurons. In the case of NPC1, cerebellar Purkinje cells are primarily affected. Of course, there are technical hurdles to obtain such cells from iPSCs in vitro, but it is surprising that hardly any research directly addresses the loss of neurons, at least for NPC1. For example, it is currently poorly understood which pathogenic mechanisms underlie the degeneration of Purkinje cells. In addition to apoptosis as the main cause of cell death, necroptosis might have a major influence on the loss of neuronal cells in NPC1. Cougnoux and colleagues [38] evaluated this possibility in NPC1-deficient fibroblasts, iPSC-derived neuronal precursor cells, and in the cerebellum of NPC1-deficient mice. They found a decreased cell viability and an increase in the receptor interacting protein kinase 1 and 3 (RIP1, RIP3) in all cell types analyzed. Both kinases are components of a protein complex, the so called necrosome, which is known to initiate necroptosis. The inhibition of RIP1 and 3 with Necrostatin-1 led to an increase in the cell viability of fibroblasts and prevented the loss of Purkinje cells in NPC1-deficient mice, prolonging their lifespan. These results suggest necroptosis as a potential target for pharmacological intervention. Sung and colleagues [49] did not analyze cell death directly, but described the defective neuronal differentiation of cells generated by the direct conversion of fibroblasts. The extent to which this may contribute to the neuropathology of NPC1 needs further clarification. However, one can be optimistic that further work will make use of iPSC models and will further contribute to the elucidation of the pathophysiological aspects of NPC1.

Studies of the pathogenic mechanisms of neurodegenerative diseases using iPSC-based models have revealed similarities in the pathology of various neurodegenerative disorders. In terms of NPC1, with an incidence of 1/120,000 live births, such similarities are described with Alzheimer’s disease, with a prevalence of 30 million people worldwide. In this regard, NPC1 and AD certainly represent an unequal pair for comparative studies, but in terms of pathogenic mechanisms there are some remarkable similarities, such as abnormal cholesterol metabolism, amyloid-β (Aβ) and tau pathology [156]. Interestingly, neurofibrillary tangles, the predominant pathophysiological feature of AD, can be observed in NPC1, and patients also show an accumulation of Aβ peptides. However, no amyloid plaques were observed, which could be explained by the fact that the vast majority of NPC1 patients die before the manifestation of amyloid plaques. It is discussed that cholesterol influences the processing of amyloid precursor protein (APP), leading to amyloid plaques by altering the endocytotic trade of APP due to a change in lipid composition in the membrane. Conversely, Alzheimer’s disease shows features of disturbed cholesterol metabolism with lipid deposits, which are the hallmark of NPC1. Interestingly, a variant of ApoE, which is the main carrier of cholesterol in the brain (apolipoprotein type 4 allele, ApoE-ε4), is one of the main risk factors for late-onset AD. 

The examples of NPC1 and AD impressively demonstrate the potential of iPSC-based disease models. It is likely that without iPSC technology it would be much more difficult to compare and elucidate pathogenic mechanisms in disease affected cell types. Certainly, comparative studies with post-mortem preparations are possible in this regard, but no insights into early phases, without clear signs of neurodegenerative processes, can be obtained from these, and, of course, longitudinal studies are not possible. It remains extremely exciting to see how future studies using iPSC-based model systems will contribute to the understanding of neurodegenerative diseases, and one may be optimistic that these findings will advance the development of new therapeutic approaches for NPC1 and neurodegenerative diseases in general.

## Figures and Tables

**Table 1:** Overview of publications.

Reference	Donor Cells Used	Reprogramming Method	Stem Cells Used	Differentiation Target(s)	Results/ Observations	Compounds Used	Genotypes Studied
**Models Based on Pluripotent Stem Cells**							
Ordonez et al. 2012 [32]	N/A	N/A	NPC1 KD hESCs	NSCs neurons	Accumulation of lysotracker positive organelles Mitochondrial fragments Increased filipin staining Defective autophagy	M-β-CD	N/A
Bergamin et al. 2013 [48]	N/A	N/A	hSKIN-MASCs	neurons	Cholesterol and GM2 accumulation Morphological changes	N/A	I1061T/I1061T I1061T/I1061T V1165M
**Models Based on Transdifferentiation**							
Sung et al. 2017 [49]	Fibroblasts	Trans-differentiation	N/A	neurons	Cholesterol accumulation Deficiencies in self-renewal of NSCs	SB202190 L-NAME valproic acid	P237S/I1061T I1061T/I1061T
**Models Based on Induced Pluripotent Stem Cells**							
Trilck et al. 2013 [11]	Fibroblasts	Retroviral	iPSCs	NPCs NDCs	Cholesterol accumulation in fibroblasts, iPSCs and NPCs	N/A	E612D/P543Rfs*20
Maetzel et al. 2014 [33]	Fibroblasts	Cre-excisable lentivirus	iPSCs	HLCs NDCs	Decreased cell viability Defects in cholesterol metabolism and autophagy	H-β-CD rapamycin bafilomycin A1 carbamazepine verapamil trehalose SMER28	I1061T/I1061T P237S/I1061T 1920delG/1009G>A
Yu et al. 2014 [34]	Fibroblasts	Sendai virus	iPSCs	NSCs neurons	Cholesterol accumulation	HP-β-CD M-β-CD δ-tocopherol	P237S/I1061T
Lee et al. 2014 [35]	Fibroblasts	Retroviral	iPSCs	neurons	Defects in VEGF signaling Defective autophagic flux	SphK inhibitor VEGF	P237S/I1061T
Soga et al. 2015 [36]	Fibroblasts	Sendai virus	iPSCs	HLCs NPCs	Cholesterol accumulation Impaired autophagy	HP-β-CD HP-γ-CD	S667L/C1161Y Y1088C/581_592delinsG
Efthymiou et al. 2015 [37]	Fibroblasts	Lentiviral	iPSCs	NSCs neurons	Impaired Ca^2+^ and Wnt3a signaling	Curcumin dantrolene BIO	I1061T/I1061T
Cougnoux et al. 2016 [38]	Fibroblasts	Sendai virus	iPSCs	NPCs	Decreased viability Increased necroptosis	Nec1	P237S/I1061T
Trilck et al. 2017 [39]	Fibroblasts	Retroviral	iPSCs	NPCs NDCs	Cholesterol and GM2 accumulation in NDCs Reduced Hex A activity	N/A	I1061T/I1061T Y394H/Y394H E612D/P543Rfs*20
Peter et al. 2017 [40]	Fibroblasts	Retroviral	iPSCs	NPCs NDCs	Methodological report of reprogramming	N/A	I1061T/I1061T Y394H/Y394H
Peter et al. 2017 [41]	Fibroblasts	Retroviral	iPSCs	NPCs NDCs	Increased number of reactive astrocytes Hypophosphorylation of GFAP and vimentin	PMA	I1061T/I1061T Y394H/Y394H E612D/P543Rfs*20
Rabenstein et al. 2017 [20]	Fibroblasts	Retroviral	iPSCs	NPCs NDCs	Decreased Ca^2+^ flux through AMPAR Increased amount of GluA2	N/A	I1061T/I1061T Y394H/Y394H E612D/P543Rfs*20
Dai et al. 2017 [42]	Fibroblasts	Sendai virus	iPSCs	NSCs neurons	Impaired macroautophagy/autophagy	HP-β-CD M-β-CD	P237S/I1061T
Li et al. 2020 [43]	Fibroblasts	Sendai virus	iPSCs	N/A	Methodological report of reprogramming	N/A	I1061T/I1061T
Völkner et al. 2020 [44]	Fibroblasts	Sendai virus	iPSCs	N/A	Methodological report of reprogramming	N/A	I1061T/I1061T
Jürs et al. 2020 [45]	Fibroblasts	Retroviral	iPSCs	NPCs NDCs	Increased level of ROS Down regulation of catalase	N/A	I1061T/I1061T Y394H/Y394H E612D/P543Rfs*20
Völkner et al. 2020 [46]	Fibroblasts	Retroviral	iPSCs	N/A	Methodological report of reprogramming	N/A	V1023Sfs*15/G992R
**Pluripotent Stem Cell-Based Models in NPC2 Disease**							
Völkner et al. 2019 [47]	Fibroblasts	Retroviral	iPSCs	N/A	Methodological report of reprogramming	N/A	E20X/C47F

N/A = not applicable.

## Abbreviations

24(S)-HC	24(S)-hydroxycholesterol
25-HC	25-hydroxycholesterol
3-MA	3-Methyladenine
AD	Alzheimer’s disease
ALS	Amyotrophic lateral sclerosis
AMPAR	AMPA-receptor
AMPK	AMP-activated protein kinase
Apo E	Apolipoprotein E
APP	Amyloid precursor protein
Cas	CRISPR-associated gene
CBZ	Carbamazepine
CD	Cyclodextrin
CNS	Central nervous system
CoQ10	Coenzyme Q10
cPCs	Cerebellar Purkinje cells
CRISPR	Clustered regularly interspaced short palindromic repeats
ER	Endoplasmic reticulum
GABAAR	GABA_A_ receptor
GD	Gaucher’s Disease
GFAP	Glial fibrillary acidic protein
GlyR	Glycine receptor
HAT	Histone acetyl transferase
HDACi	Histone deacetylase inhibitor
hESC	Human embryonic stem cell
Hex A	Hexosaminidase A
HLC	Hepatocyte-like cell
hSKIN-MASCS	Human skin multipotent adult stem cells
HP-α-CD	Hydroxypropyl-alpha-cyclodextrin
HP-β-CD	Hydroxypropyl-beta-cyclodextrin
HP-γ-CD	Hydroxypropyl-gamma-cyclodextrin
HSEM	Horizontal saccadic eye movement
iPSC	Induced pluripotent stem cell
KD	Knock down
LC3B	Microtubule-associated protein a light chain 3B
LE/LY	Late endosome/lysosome
LSD	Lysosomal storage disorder
M-β-CD	Methyl-beta-cyclodextrin
NDCs	Neuronal differentiated cells
NPC	Neural progenitor cell
NPC1	Niemann-Pick type C1
NPC2	Niemann-Pick type C2
NSS	Neurological Severity Scores
OS	Oxidative stress
PC	Pharmacological chaperone
PD	Parkinson’s disease
PMA	Phorbol 12-myristate 13-acetate
PSCs	Pluripotent stem cells
Rap	Rapamycin
RIP1/3	Receptor interacting protein kinase 1/3
ROS	Reactive oxygen species
S1P	Sphingosine-1-phosphate
SAHA	Suberanilohydroxamic acid
SOD	Superoxide-Dismutase
SphK	Sphingosine kinase
SRT	Substrate reduction therapy
TALEN	Transcription activator-like effector nuclease
Tre	Trehalose
VEGF	Vascular endothelial growth factor
Ver	Verapamil
VSGP	Vertical supranuclear gaze palsy

## References

[1] Vanier M.T. (2010). Niemann-Pick disease type C. Orphanet J. Rare Dis..

[2] The Human Gene Mutation Database. Gene Database. http://www.hgmd.cf.ac.uk/ac/index.php.

[3] Wraith J.E., Baumgartner M.R., Bembi B., Covanis A., Levade T., Mengel E., Pineda M., Sedel F., Topçu M., Vanier M.T. (2009). Recommendations on the diagnosis and management of Niemann-Pick disease type C. Mol. Genet. Metab..

[4] Carstea E.D., Morris J.A., Coleman K.G., Loftus S.K., Zhang D., Cummings C., Gu J., Rosenfeld M.A., Pavan W.J., Krizman D.B. (1997). Niemann-Pick C1 disease gene: Homology to mediators of cholesterol homeostasis. Science.

[5] Park I.-H., Arora N., Huo H., Maherali N., Ahfeldt T., Shimamura A., Lensch M.W., Cowan C., Hochedlinger K., Daley G.Q. (2008). Disease-specific induced pluripotent stem cells. Cell.

[6] Kawagoe S., Higuchi T., Meng X.-L., Shimada Y., Shimizu H., Hirayama R., Fukuda T., Chang H., Nakahata T., Fukada S. (2011). Generation of induced pluripotent stem (iPS) cells derived from a murine model of Pompe disease and differentiation of Pompe-iPS cells into skeletal muscle cells. Mol. Genet. Metab..

[7] Doerr J., Böckenhoff A., Ewald B., Ladewig J., Eckhardt M., Gieselmann V., Matzner U., Brüstle O., Koch P. (2015). Arylsulfatase A Overexpressing Human iPSC-derived Neural Cells Reduce CNS Sulfatide Storage in a Mouse Model of Metachromatic Leukodystrophy. Mol. Ther..

[8] Lojewski X., Staropoli J.F., Biswas-Legrand S., Simas A.M., Haliw L., Selig M.K., Coppel S.H., Goss K.A., Petcherski A., Chandrachud U. (2014). Human iPSC models of neuronal ceroid lipofuscinosis capture distinct effects of TPP1 and CLN3 mutations on the endocytic pathway. Hum. Mol. Genet..

[9] Tolar J., Park I.-H., Xia L., Lees C.J., Peacock B., Webber B., McElmurry R.T., Eide C.R., Orchard P.J., Kyba M. (2011). Hematopoietic differentiation of induced pluripotent stem cells from patients with mucopolysaccharidosis type I (Hurler syndrome). Blood.

[10] Long Y., Xu M., Li R., Dai S., Beers J., Chen G., Soheilian F., Baxa U., Wang M., Marugan J.J. (2016). Induced Pluripotent Stem Cells for Disease Modeling and Evaluation of Therapeutics for Niemann-Pick Disease Type A. Stem Cells Transl. Med..

[11] Trilck M., Hübner R., Seibler P., Klein C., Rolfs A., Frech M.J. (2013). Niemann-Pick type C1 patient-specific induced pluripotent stem cells display disease specific hallmarks. Orphanet J. Rare Dis..

[12] Fog C.K., Kirkegaard T. (2019). Animal models for Niemann-Pick type C: Implications for drug discovery & development. Expert Opin. Drug Discov..

[13] Pallottini V., Pfrieger F.W. (2020). Understanding and Treating Niemann-Pick Type C Disease: Models Matter. Int. J. Mol. Sci..

[14] Chiba Y., Komori H., Takei S., Hasegawa-Ishii S., Kawamura N., Adachi K., Nanba E., Hosokawa M., Enokido Y., Kouchi Z. (2014). Niemann-Pick disease type C1 predominantly involving the frontotemporal region, with cortical and brainstem Lewy bodies: An autopsy case. Neuropathology.

[15] Cologna S.M., Cluzeau C.V.M., Yanjanin N.M., Blank P.S., Dail M.K., Siebel S., Toth C.L., Wassif C.A., Lieberman A.P., Porter F.D. (2014). Human and mouse neuroinflammation markers in Niemann-Pick disease, type C1. J. Inherit. Metab. Dis..

[16] Pentchev P.G., Gal A.E., Booth A.D., Omodeo-Sale F., Fours J., Neumeyer B.A., Quirk J.M., Dawson G., Brady R.O. (1980). A lysosomal storage disorder in mice characterized by a dual deficiency of sphingomyelinase and glucocerebrosidase. Biochim. Biophys. Acta (BBA)-Lipids Lipid Metab..

[17] Võikar V., Rauvala H., Ikonen E. (2002). Cognitive deficit and development of motor impairment in a mouse model of Niemann-Pick type C disease. Behav. Brain Res..

[18] Morris M.D., Bhuvaneswaran C., Shio H., Fowler S. (1982). Lysosome lipid storage disorder in NCTR-BALB/c mice. I. Description of the disease and genetics. Am. J. Pathol..

[19] Rabenstein M., Peter F., Rolfs A., Frech M.J. (2018). Impact of Reduced Cerebellar EAAT Expression on Purkinje Cell Firing Pattern of NPC1-deficient Mice. Sci. Rep..

[20] Rabenstein M., Peter F., Joost S., Trilck M., Rolfs A., Frech M.J. (2017). Decreased calcium flux in Niemann-Pick type C1 patient-specific iPSC-derived neurons due to higher amount of calcium-impermeable AMPA receptors. Mol. Cell. Neurosci..

[21] German D.C., Liang C.L., Song T., Yazdani U., Xie C., Dietschy J.M. (2002). Neurodegeneration in the Niemann-Pick C mouse: Glial involvement. Neuroscience.

[22] Brown D.E., Thrall M.A., Walkley S.U., Wenger D.A., Mitchell T.W., Smith M.O., Royals K.L., March P.A., Allison R.W. (1994). Feline Niemann-Pick disease type C. Am. J. Pathol..

[23] Walkley S.U., Suzuki K. (2004). Consequences of NPC1 and NPC2 loss of function in mammalian neurons. Biochim. Biophys. Acta.

[24] Miyawaki S., Mitsuoka S., Sakiyama T., Kitagawa T. (1982). Sphingomyelinosis, a new mutation in the mouse: A model of Niemann-Pick disease in humans. J. Hered..

[25] Xie X., Brown M.S., Shelton J.M., Richardson J.A., Goldstein J.L., Liang G. (2011). Amino acid substitution in NPC1 that abolishes cholesterol binding reproduces phenotype of complete NPC1 deficiency in mice. Proc. Natl. Acad. Sci. USA.

[26] Praggastis M., Tortelli B., Zhang J., Fujiwara H., Sidhu R., Chacko A., Chen Z., Chung C., Lieberman A.P., Sikora J. (2015). A murine Niemann-Pick C1 I1061T knock-in model recapitulates the pathological features of the most prevalent human disease allele. J. Neurosci..

[27] Maue R.A., Burgess R.W., Wang B., Wooley C.M., Seburn K.L., Vanier M.T., Rogers M.A., Chang C.C., Chang T.-Y., Harris B.T. (2012). A novel mouse model of Niemann-Pick type C disease carrying a D1005G-Npc1 mutation comparable to commonly observed human mutations. Hum. Mol. Genet..

[28] Saito Y., Suzuki K., Nanba E., Yamamoto T., Ohno K., Murayama S. (2002). Niemann-Pick type C disease: Accelerated neurofibrillary tangle formation and amyloid beta deposition associated with apolipoprotein E epsilon 4 homozygosity. Ann. Neurol..

[29] Lopez M.E., Scott M.P. (2013). Genetic dissection of a cell-autonomous neurodegenerative disorder: Lessons learned from mouse models of Niemann-Pick disease type C. Dis. Model. Mech..

[30] Singh V.K., Kalsan M., Kumar N., Saini A., Chandra R. (2015). Induced pluripotent stem cells: Applications in regenerative medicine, disease modeling, and drug discovery. Front. Cell Dev. Biol..

[31] Omole A.E., Fakoya A.O.J. (2018). Ten years of progress and promise of induced pluripotent stem cells: Historical origins, characteristics, mechanisms, limitations, and potential applications. PeerJ.

[32] Ordonez M.P., Roberts E.A., Kidwell C.U., Yuan S.H., Plaisted W.C., Goldstein L.S.B. (2012). Disruption and therapeutic rescue of autophagy in a human neuronal model of Niemann Pick type C1. Hum. Mol. Genet..

[33] Maetzel D., Sarkar S., Wang H., Abi-Mosleh L., Xu P., Cheng A.W., Gao Q., Mitalipova M., Jaenisch R. (2014). Genetic and chemical correction of cholesterol accumulation and impaired autophagy in hepatic and neural cells derived from Niemann-Pick Type C patient-specific iPS cells. Stem Cell Rep..

[34] Yu D., Swaroop M., Wang M., Baxa U., Yang R., Yan Y., Coksaygan T., DeTolla L., Marugan J.J., Austin C.P. (2014). Niemann-Pick Disease Type C: Induced Pluripotent Stem Cell-Derived Neuronal Cells for Modeling Neural Disease and Evaluating Drug Efficacy. J. Biomol. Screen..

[35] Lee H., Lee J.K., Park M.H., Hong Y.R., Marti H.H., Kim H., Okada Y., Otsu M., Seo E.-J., Park J.-H. (2014). Pathological roles of the VEGF/SphK pathway in Niemann-Pick type C neurons. Nat. Commun..

[36] Soga M., Ishitsuka Y., Hamasaki M., Yoneda K., Furuya H., Matsuo M., Ihn H., Fusaki N., Nakamura K., Nakagata N. (2015). HPGCD outperforms HPBCD as a potential treatment for Niemann-Pick disease type C during disease modeling with iPS cells. Stem Cells.

[37] Efthymiou A.G., Steiner J., Pavan W.J., Wincovitch S., Larson D.M., Porter F.D., Rao M.S., Malik N. (2015). Rescue of an in vitro neuron phenotype identified in Niemann-Pick disease, type C1 induced pluripotent stem cell-derived neurons by modulating the WNT pathway and calcium signaling. Stem Cells Transl. Med..

[38] Cougnoux A., Cluzeau C., Mitra S., Li R., Williams I., Burkert K., Xu X., Wassif C.A., Zheng W., Porter F.D. (2016). Necroptosis in Niemann-Pick disease, type C1: A potential therapeutic target. Cell Death Dis..

[39] Trilck M., Peter F., Zheng C., Frank M., Dobrenis K., Mascher H., Rolfs A., Frech M.J. (2017). Diversity of glycosphingolipid GM2 and cholesterol accumulation in NPC1 patient-specific iPSC-derived neurons. Brain Res..

[40] Peter F., Trilck M., Rabenstein M., Rolfs A., Frech M.J. (2017). Dataset in support of the generation of Niemann-Pick disease Type C1 patient-specific iPS cell lines carrying the novel NPC1 mutation c.1180TC or the prevalent c.3182TC mutation-Analysis of pluripotency and neuronal differentiation. Data Brief.

[41] Peter F., Rost S., Rolfs A., Frech M.J. (2017). Activation of PKC triggers rescue of NPC1 patient specific iPSC derived glial cells from gliosis. Orphanet J. Rare Dis..

[42] Dai S., Dulcey A.E., Hu X., Wassif C.A., Porter F.D., Austin C.P., Ory D.S., Marugan J., Zheng W. (2017). Methyl-β-cyclodextrin restores impaired autophagy flux in Niemann-Pick C1-deficient cells through activation of AMPK. Autophagy.

[43] Li R., Pradhan M., Xu M., Roeder A., Beers J., Zou J., Liu C., Porter F.D., Zheng W. (2020). An induced pluripotent stem cell line (TRNDi001-D) from a Niemann-Pick disease type C1 (NPC1) patient carrying a homozygous p. I1061T (c. 3182TC) mutation in the NPC1 gene. Stem Cell Res..

[44] Völkner C., Liedtke M., Petters J., Huth K., Knuebel G., Murua Escobar H., Bullerdiek J., Lukas J., Hermann A., Frech M.J. (2020). Generation of an iPSC line (AKOSi006-A) from fibroblasts of a NPC1 patient, carrying the homozygous mutation p.I1061T (c.3182 T C) and a control iPSC line (AKOSi007-A) using a non-integrating Sendai virus system. Stem Cell Res..

[45] Jürs A.V., Völkner C., Liedtke M., Huth K., Lukas J., Hermann A., Frech M.J. (2020). Oxidative Stress and Alterations in the Antioxidative Defense System in Neuronal Cells Derived from NPC1 Patient-Specific Induced Pluripotent Stem Cells. Int. J. Mol. Sci..

[46] Völkner C., Liedtke M., Petters J., Lukas J., Murua Escobar H., Knuebel G., Bullerdiek J., Holzmann C., Hermann A., Frech M.J. (2021). Generation of an iPSC line (AKOSi004-A) from fibroblasts of a female adult NPC1 patient, carrying the compound heterozygous mutation p.Val1023Serfs*15/p.Gly992Arg and of an iPSC line (AKOSi005-A) from a female adult control individual. Stem Cell Res..

[47] Völkner C., Peter F., Liedtke M., Krohn S., Lindner I., Murua Escobar H., Cimmaruta C., Lukas J., Hermann A., Frech M.J. (2019). Generation of the Niemann-Pick type C2 patient-derived iPSC line AKOSi001-A. Stem Cell Res..

[48] Bergamin N., Dardis A., Beltrami A., Cesselli D., Rigo S., Zampieri S., Domenis R., Bembi B., Beltrami C.A. (2013). A human neuronal model of Niemann Pick C disease developed from stem cells isolated from patient’s skin. Orphanet J. Rare Dis..

[49] Sung E.-A., Yu K.-R., Shin J.-H., Seo Y., Kim H.-S., Guen Koog M., Kang I., Kim J.-J., Lee B.-C., Shin T.-H. (2017). Generation of patient specific human neural stem cells from Niemann-Pick disease type C patient-derived fibroblasts. Oncotarget.

[50] Bräuer A.U., Kuhla A., Holzmann C., Wree A., Witt M. (2019). Current Challenges in Understanding the Cellular and Molecular Mechanisms in Niemann-Pick Disease Type C1. Int. J. Mol. Sci..

[51] Maxfield F.R., Wüstner D. (2012). Analysis of cholesterol trafficking with fluorescent probes. Methods Cell Biol..

[52] Millat G., Marçais C., Tomasetto C., Chikh K., Fensom A.H., Harzer K., Wenger D.A., Ohno K., Vanier M.T. (2001). Niemann-Pick C1 disease: Correlations between NPC1 mutations, levels of NPC1 protein, and phenotypes emphasize the functional significance of the putative sterol-sensing domain and of the cysteine-rich luminal loop. Am. J. Hum. Genet..

[53] Sun X., Marks D.L., Park W.D., Wheatley C.L., Puri V., O’Brien J.F., Kraft D.L., Lundquist P.A., Patterson M.C., Pagano R.E. (2001). Niemann-Pick C variant detection by altered sphingolipid trafficking and correlation with mutations within a specific domain of NPC1. Am. J. Hum. Genet..

[54] Barton B.R., Kompoliti K., Verhagen L. (2010). Niemann–Pick Type C. Encyclopedia of Movement Disorders.

[55] Genaro-Mattos T.C., Anderson A., Allen L.B., Korade Z., Mirnics K. (2019). Cholesterol Biosynthesis and Uptake in Developing Neurons. ACS Chem. Neurosci..

[56] Pfrieger F.W., Ungerer N. (2011). Cholesterol metabolism in neurons and astrocytes. Prog. Lipid Res..

[57] Vanier M.T. (1999). Lipid changes in Niemann-Pick disease type C brain: Personal experience and review of the literature. Neurochem. Res..

[58] Yano T., Taniguchi M., Akaboshi S., Vanier M.T., Tai T., Sakuraba H., Ohno K. (1996). Accumulation of GM2 Ganglioside Accumulation of GM2 in Niemann-Pick Type C Fibroblasts. Proc. Jpn. Acad..

[59] Taniguchi M., Shinoda Y., Ninomiya H., Vanier M.T., Ohno K. (2001). Sites and temporal changes of gangliosides GM1/GM2 storage in the Niemann-Pick disease type C mouse brain. Brain Dev..

[60] Jiménez-Moreno N., Stathakos P., Caldwell M.A., Lane J.D. (2017). Induced Pluripotent Stem Cell Neuronal Models for the Study of Autophagy Pathways in Human Neurodegenerative Disease. Cells.

[61] Füllgrabe J., Klionsky D.J., Joseph B. (2013). Histone post-translational modifications regulate autophagy flux and outcome. Autophagy.

[62] Loos B., Du Toit A., Hofmeyr J.-H.S. (2014). Defining and measuring autophagosome flux—concept and reality. Autophagy.

[63] Kabeya Y., Mizushima N., Ueno T., Yamamoto A., Kirisako T., Noda T., Kominami E., Ohsumi Y., Yoshimori T. (2000). LC3, a mammalian homologue of yeast Apg8p, is localized in autophagosome membranes after processing. EMBO J..

[64] Singh R., Kaushik S., Wang Y., Xiang Y., Novak I., Komatsu M., Tanaka K., Cuervo A.M., Czaja M.J. (2009). Autophagy regulates lipid metabolism. Nature.

[65] Nixon R.A., Wegiel J., Kumar A., Yu W.H., Peterhoff C., Cataldo A., Cuervo A.M. (2005). Extensive involvement of autophagy in Alzheimer disease: An immuno-electron microscopy study. J. Neuropathol. Exp. Neurol..

[66] Anglade P., Vyas S., Javoy-Agid F., Herrero M.T., Michel P.P., Marquez J., Mouatt-Prigent A., Ruberg M., Hirsch E.C., Agid Y. (1997). Apoptosis and autophagy in nigral neurons of patients with Parkinson’s disease. Histol. Histopathol..

[67] Martinez-Vicente M., Talloczy Z., Wong E., Tang G., Koga H., Kaushik S., de Vries R., Arias E., Harris S., Sulzer D. (2010). Cargo recognition failure is responsible for inefficient autophagy in Huntington’s disease. Nat. Neurosci..

[68] Song C., Guo J., Liu Y., Tang B. (2012). Autophagy and Its Comprehensive Impact on ALS. Int. J. Neurosci..

[69] Ko D.C., Milenkovic L., Beier S.M., Manuel H., Buchanan J., Scott M.P. (2005). Cell-autonomous death of cerebellar purkinje neurons with autophagy in Niemann-Pick type C disease. PLoS Genet..

[70] Liao G., Yao Y., Liu J., Yu Z., Cheung S., Xie A., Liang X., Bi X. (2007). Cholesterol accumulation is associated with lysosomal dysfunction and autophagic stress in Npc1 -/- mouse brain. Am. J. Pathol..

[71] Pacheco C.D., Lieberman A.P. (2007). Lipid trafficking defects increase Beclin-1 and activate autophagy in Niemann-Pick type C disease. Autophagy.

[72] Pacheco C.D., Kunkel R., Lieberman A.P. (2007). Autophagy in Niemann-Pick C disease is dependent upon Beclin-1 and responsive to lipid trafficking defects. Hum. Mol. Genet..

[73] Evans C.S., Holzbaur E.L.F. (2019). Autophagy and mitophagy in ALS. Neurobiol. Dis..

[74] Uddin M.S., Stachowiak A., Mamun A.A., Tzvetkov N.T., Takeda S., Atanasov A.G., Bergantin L.B., Abdel-Daim M.M., Stankiewicz A.M. (2018). Autophagy and Alzheimer’s Disease: From Molecular Mechanisms to Therapeutic Implications. Front. Aging Neurosci..

[75] Moors T.E., Hoozemans J.J.M., Ingrassia A., Beccari T., Parnetti L., Chartier-Harlin M.-C., van de Berg W.D.J. (2017). Therapeutic potential of autophagy-enhancing agents in Parkinson’s disease. Mol. Neurodegener..

[76] Saxton R.A., Sabatini D.M. (2017). mTOR Signaling in Growth, Metabolism, and Disease. Cell.

[77] Yan M.H., Wang X., Zhu X. (2013). Mitochondrial defects and oxidative stress in Alzheimer disease and Parkinson disease. Free Radic. Biol. Med..

[78] Fu R., Yanjanin N.M., Bianconi S., Pavan W.J., Porter F.D. (2010). Oxidative stress in Niemann-Pick disease, type C. Mol. Genet. Metab..

[79] Wu Y., Chen M., Jiang J. (2019). Mitochondrial dysfunction in neurodegenerative diseases and drug targets via apoptotic signaling. Mitochondrion.

[80] Elfawy H.A., Das B. (2019). Crosstalk between mitochondrial dysfunction, oxidative stress, and age related neurodegenerative disease: Etiologies and therapeutic strategies. Life Sci..

[81] Ordonez M.P. (2012). Defective mitophagy in human Niemann-Pick Type C1 neurons is due to abnormal autophagy activation. Autophagy.

[82] Yu W., Gong J.-S., Ko M., Garver W.S., Yanagisawa K., Michikawa M. (2005). Altered cholesterol metabolism in Niemann-Pick type C1 mouse brains affects mitochondrial function. J. Biol. Chem..

[83] Marí M., Caballero F., Colell A., Morales A., Caballeria J., Fernandez A., Enrich C., Fernandez-Checa J.C., García-Ruiz C. (2006). Mitochondrial free cholesterol loading sensitizes to TNF- and Fas-mediated steatohepatitis. Cell Metab..

[84] Vázquez M.C., Balboa E., Alvarez A.R., Zanlungo S. (2012). Oxidative stress: A pathogenic mechanism for Niemann-Pick type C disease. Oxidative Med. Cell. Longev..

[85] Fernández A., Llacuna L., Fernández-Checa J.C., Colell A. (2009). Mitochondrial cholesterol loading exacerbates amyloid beta peptide-induced inflammation and neurotoxicity. J. Neurosci..

[86] Zampieri S., Mellon S.H., Butters T.D., Nevyjel M., Covey D.F., Bembi B., Dardis A. (2009). Oxidative stress in NPC1 deficient cells: Protective effect of allopregnanolone. J. Cell. Mol. Med..

[87] Cologna S.M., Jiang X.-S., Backlund P.S., Cluzeau C.V.M., Dail M.K., Yanjanin N.M., Siebel S., Toth C.L., Jun H., Wassif C.A. (2012). Quantitative proteomic analysis of Niemann-Pick disease, type C1 cerebellum identifies protein biomarkers and provides pathological insight. PLoS ONE.

[88] Suzuki H., Sakiyama T., Harada N., Abe M., Tadokoro M. (2003). Pathologic changes of glial cells in murine model of Niemann-Pick disease type C: Immunohistochemical, lectin-histochemical and ultrastructural observations. Pediatr. Int..

[89] Tamari F., Chen F.W., Li C., Chaudhari J., Ioannou Y.A. (2013). PKC activation in Niemann pick C1 cells restores subcellular cholesterol transport. PLoS.ONE..

[90] Walter M., Chen F.W., Tamari F., Wang R., Ioannou Y.A. (2009). Endosomal lipid accumulation in NPC1 leads to inhibition of PKC, hypophosphorylation of vimentin and Rab9 entrapment. Biol. Cell.

[91] Ioannou Y.A., Altstiel L., Crockford D.R., Kongsamut M. (2017). Methods and Compositions for Treatment of Lipid Storage Disorders. U.S. Patent.

[92] Pentchev P.G., Boothe A.D., Kruth H.S., Weintroub H., Stivers J., Brady R.O. (1984). A genetic storage disorder in BALB/C mice with a metabolic block in esterification of exogenous cholesterol. J. Biol. Chem..

[93] Davidson C.D., Ali N.F., Micsenyi M.C., Stephney G., Renault S., Dobrenis K., Ory D.S., Vanier M.T., Walkley S.U. (2009). Chronic cyclodextrin treatment of murine Niemann-Pick C disease ameliorates neuronal cholesterol and glycosphingolipid storage and disease progression. PLoS ONE.

[94] Sarna J.R., Larouche M., Marzban H., Sillitoe R.V., Rancourt D.E., Hawkes R. (2003). Patterned Purkinje cell degeneration in mouse models of Niemann-Pick type C disease. J. Comp. Neurol..

[95] Perkins E., Suminaite D., Jackson M. (2016). Cerebellar ataxias: Β-III spectrin’s interactions suggest common pathogenic pathways. J. Physiol..

[96] Rabenstein M., Murr N., Hermann A., Rolfs A., Frech M.J. (2019). Alteration of GABAergic Input Precedes Neurodegeneration of Cerebellar Purkinje Cells of NPC1-Deficient Mice. Int. J. Mol. Sci..

[97] Feng X., Yang F., Rabenstein M., Wang Z., Frech M.J., Wree A., Bräuer A.U., Witt M., Gläser A., Hermann A. (2020). Stimulation of mGluR1/5 Improves Defective Internalization of AMPA Receptors in NPC1 Mutant Mouse. Cereb. Cortex.

[98] Feng X., Bader B.M., Yang F., Segura M., Schultz L., Schröder O.H.-U., Rolfs A., Luo J. (2018). Improvement of impaired electrical activity in NPC1 mutant cortical neurons upon DHPG stimulation detected by micro-electrode array. Brain Res..

[99] Elstein D., Hollak C., Aerts J.M.F.G., van Weely S., Maas M., Cox T.M., Lachmann R.H., Hrebicek M., Platt F.M., Butters T.D. (2004). Sustained therapeutic effects of oral miglustat (Zavesca, N-butyldeoxynojirimycin, OGT 918) in type I Gaucher disease. J. Inherit. Metab. Dis..

[100] Wraith J.E., Imrie J. (2009). New therapies in the management of Niemann-Pick type C disease: Clinical utility of miglustat. Ther. Clin. Risk Manag..

[101] Lyseng-Williamson K.A. (2014). Miglustat: A review of its use in Niemann-Pick disease type C. Drugs.

[102] Zervas M., Somers K.L., Thrall M.A., Walkley S.U. (2001). Critical role for glycosphingolipids in Niemann-Pick disease type C. Curr. Biol..

[103] Patterson M.C., Vecchio D., Prady H., Abel L., Wraith J.E. (2007). Miglustat for treatment of Niemann-Pick C disease: A randomised controlled study. Lancet Neurol..

[104] Wraith J.E., Vecchio D., Jacklin E., Abel L., Chadha-Boreham H., Luzy C., Giorgino R., Patterson M.C. (2010). Miglustat in adult and juvenile patients with Niemann-Pick disease type C: Long-term data from a clinical trial. Mol. Genet. Metab..

[105] Patterson M.C., Vecchio D., Jacklin E., Abel L., Chadha-Boreham H., Luzy C., Giorgino R., Wraith J.E. (2010). Long-term miglustat therapy in children with Niemann-Pick disease type C. J. Child Neurol..

[106] Pineda M., Wraith J.E., Mengel E., Sedel F., Hwu W.-L., Rohrbach M., Bembi B., Walterfang M., Korenke G.C., Marquardt T. (2009). Miglustat in patients with Niemann-Pick disease Type C (NP-C): A multicenter observational retrospective cohort study. Mol. Genet. Metab..

[107] Jansook P., Ogawa N., Loftsson T. (2018). Cyclodextrins: Structure, physicochemical properties and pharmaceutical applications. Int. J. Pharm..

[108] Szente L., Szejtli J. (2004). Cyclodextrins as food ingredients. Trends Food Sci. Technol..

[109] Del Valle E. (2004). Cyclodextrins and their uses: A review. Process Biochem..

[110] Ottinger E.A., Kao M.L., Carrillo-Carrasco N., Yanjanin N., Shankar R.K., Janssen M., Brewster M., Scott I., Xu X., Cradock J. (2014). Collaborative development of 2-hydroxypropyl-β-cyclodextrin for the treatment of Niemann-Pick type C1 disease. Curr. Top. Med. Chem..

[111] Griffin L.D., Gong W., Verot L., Mellon S.H. (2004). Niemann-Pick type C disease involves disrupted neurosteroidogenesis and responds to allopregnanolone. Nat. Med..

[112] Liu B., Turley S.D., Burns D.K., Miller A.M., Repa J.J., Dietschy J.M. (2009). Reversal of defective lysosomal transport in NPC disease ameliorates liver dysfunction and neurodegeneration in the npc1-/- mouse. Proc. Natl. Acad. Sci. USA.

[113] Liu B., Ramirez C.M., Miller A.M., Repa J.J., Turley S.D., Dietschy J.M. (2010). Cyclodextrin overcomes the transport defect in nearly every organ of NPC1 mice leading to excretion of sequestered cholesterol as bile acid. J. Lipid Res..

[114] Pontikis C.C., Davidson C.D., Walkley S.U., Platt F.M., Begley D.J. (2013). Cyclodextrin alleviates neuronal storage of cholesterol in Niemann-Pick C disease without evidence of detectable blood-brain barrier permeability. J. Inherit. Metab. Dis..

[115] Rosenbaum A.I., Zhang G., Warren J.D., Maxfield F.R. (2010). Endocytosis of beta-cyclodextrins is responsible for cholesterol reduction in Niemann-Pick type C mutant cells. Proc. Natl. Acad. Sci. USA.

[116] Ory D.S., Ottinger E.A., Farhat N.Y., King K.A., Jiang X., Weissfeld L., Berry-Kravis E., Davidson C.D., Bianconi S., Keener L.A. (2017). Intrathecal 2-hydroxypropyl-β-cyclodextrin decreases neurological disease progression in Niemann-Pick disease, type C1: A non-randomised, open-label, phase 1–2 trial. Lancet.

[117] Farmer C.A., Thurm A., Farhat N., Bianconi S., Keener L.A., Porter F.D. (2019). Long-term neuropsychological outcomes from an open-label phase 1/2a trial of 2-hydoxypropyl-β-cyclodextrins (VTS-270) in Niemann-Pick Disease, Type C1. CNS Drugs.

[118] Davidson C.D., Fishman Y.I., Puskás I., Szemán J., Sohajda T., McCauliff L.A., Sikora J., Storch J., Vanier M.T., Szente L. (2016). Efficacy and ototoxicity of different cyclodextrins in Niemann-Pick C disease. Ann. Clin. Transl. Neurol..

[119] Peterson C.L. (2002). HDAC’s at Work. Mol. Cell.

[120] Kawai H., Li H., Avraham S., Jiang S., Avraham H.K. (2003). Overexpression of histone deacetylase HDAC1 modulates breast cancer progression by negative regulation of estrogen receptor alpha. Int. J. Cancer.

[121] Sugars K.L., Rubinsztein D.C. (2003). Transcriptional abnormalities in Huntington disease. Trends Genet..

[122] Kim S.-J., Lee B.-H., Lee Y.-S., Kang K.-S. (2007). Defective cholesterol traffic and neuronal differentiation in neural stem cells of Niemann-Pick type C disease improved by valproic acid, a histone deacetylase inhibitor. Biochem. Biophys. Res. Commun..

[123] Munkacsi A.B., Chen F.W., Brinkman M.A., Higaki K., Gutiérrez G.D., Chaudhari J., Layer J.V., Tong A., Bard M., Boone C. (2011). An “Exacerbate-reverse” Strategy in Yeast Identifies Histone Deacetylase Inhibition as a Correction for Cholesterol and Sphingolipid Transport Defects in Human Niemann-Pick Type C Disease*. J. Biol. Chem..

[124] Pipalia N.H., Cosner C.C., Huang A., Chatterjee A., Bourbon P., Farley N., Helquist P., Wiest O., Maxfield F.R. (2011). Histone deacetylase inhibitor treatment dramatically reduces cholesterol accumulation in Niemann-Pick type C1 mutant human fibroblasts. Proc. Natl. Acad. Sci. USA.

[125] Hait N.C., Allegood J., Maceyka M., Strub G.M., Harikumar K.B., Singh S.K., Luo C., Marmorstein R., Kordula T., Milstien S. (2009). Regulation of histone acetylation in the nucleus by sphingosine-1-phosphate. Science.

[126] Parenti G. (2009). Treating lysosomal storage diseases with pharmacological chaperones: From concept to clinics. EMBO Mol. Med..

[127] Valenzano K.J., Khanna R., Powe A.C., Boyd R., Lee G., Flanagan J.J., Benjamin E.R. (2011). Identification and characterization of pharmacological chaperones to correct enzyme deficiencies in lysosomal storage disorders. ASSAY Drug Dev. Technol..

[128] Millat G., Marcais C., Rafi M.A., Yamamoto T., Morris J.A., Pentchev P.G., Ohno K., Wenger D.A., Vanier M.T. (1999). Niemann-Pick C1 disease: The I1061T substitution is a frequent mutant allele in patients of Western European descent and correlates with a classic juvenile phenotype. Am. J. Hum. Genet..

[129] Gelsthorpe M.E., Baumann N., Millard E., Gale S.E., Langmade S.J., Schaffer J.E., Ory D.S. (2008). Niemann-Pick type C1 I1061T mutant encodes a functional protein that is selected for endoplasmic reticulum-associated degradation due to protein misfolding. J. Biol. Chem..

[130] Ohgane K., Karaki F., Dodo K., Hashimoto Y. (2013). Discovery of oxysterol-derived pharmacological chaperones for NPC1: Implication for the existence of second sterol-binding site. Chem. Biol..

[131] Fukuda H., Karaki F., Dodo K., Noguchi-Yachide T., Ishikawa M., Hashimoto Y., Ohgane K. (2017). Phenanthridin-6-one derivatives as the first class of non-steroidal pharmacological chaperones for Niemann-Pick disease type C1 protein. Bioorganic Med. Chem. Lett..

[132] Takahashi K., Yamanaka S. (2006). Induction of Pluripotent Stem Cells from Mouse Embryonic and Adult Fibroblast Cultures by Defined Factors. Cell.

[133] Takahashi K., Tanabe K., Ohnuki M., Narita M., Ichisaka T., Tomoda K., Yamanaka S. (2007). Induction of Pluripotent Stem Cells from Adult Human Fibroblasts by Defined Factors. Cell.

[134] Yu J., Vodyanik M.A., Smuga-Otto K., Antosiewicz-Bourget J., Frane J.L., Tian S., Nie J., Jonsdottir G.A., Ruotti V., Stewart R. (2007). Induced pluripotent stem cell lines derived from human somatic cells. Science.

[135] González F., Boué S., Belmonte J.C.I. (2011). Methods for making induced pluripotent stem cells: Reprogramming à la carte. Nat. Rev. Genet..

[136] Aasen T., Raya A., Barrero M.J., Garreta E., Consiglio A., Gonzalez F., Vassena R., Bilić J., Pekarik V., Tiscornia G. (2008). Efficient and rapid generation of induced pluripotent stem cells from human keratinocytes. Nat. Biotechnol..

[137] Su R.J., Neises A., Zhang X.-B., Turksen K., Nagy A. (2016). Generation of iPS Cells from Human Peripheral Blood Mononuclear Cells Using Episomal Vectors. Induced Pluripotent Stem (iPS) Cells: Methods and Protocols.

[138] Steinle H., Weber M., Behring A., Mau-Holzmann U., von Ohle C., Popov A.-F., Schlensak C., Wendel H.P., Avci-Adali M. (2019). Reprogramming of Urine-Derived Renal Epithelial Cells into iPSCs Using srRNA and Consecutive Differentiation into Beating Cardiomyocytes. Mol. Ther.-Nucleic Acids.

[139] Ben-Ari Y. (2014). The GABA excitatory/inhibitory developmental sequence: A personal journey. Neuroscience.

[140] Miller J.D., Ganat Y.M., Kishinevsky S., Bowman R.L., Liu B., Tu E.Y., Mandal P.K., Vera E., Shim J., Kriks S. (2013). Human iPSC-based modeling of late-onset disease via progerin-induced aging. Cell Stem Cell.

[141] Lopez-Leon M., Reggiani P.C., Herenu C.B., Goya R.G. (2014). Regenerative Medicine for the Aging Brain. Enliven. J. Stem Cell Res. Regen. Med..

[142] Hou S., Lu P. (2016). Direct reprogramming of somatic cells into neural stem cells or neurons for neurological disorders. Neural Regen. Res..

[143] Papapetrou E.P., Tomishima M.J., Chambers S.M., Mica Y., Reed E., Menon J., Tabar V., Mo Q., Studer L., Sadelain M. (2009). Stoichiometric and temporal requirements of Oct4, Sox2, Klf4, and c-Myc expression for efficient human iPSC induction and differentiation. Proc. Natl. Acad. Sci. USA.

[144] Zhou W., Freed C.R. (2009). Adenoviral gene delivery can reprogram human fibroblasts to induced pluripotent stem cells. Stem Cells.

[145] Fusaki N., Ban H., Nishiyama A., Saeki K., Hasegawa M. (2009). Efficient induction of transgene-free human pluripotent stem cells using a vector based on Sendai virus, an RNA virus that does not integrate into the host genome. Proc. Jpn. Acad. Ser. B.

[146] Okita K., Nakagawa M., Hyenjong H., Ichisaka T., Yamanaka S. (2008). Generation of mouse induced pluripotent stem cells without viral vectors. Science.

[147] Si-Tayeb K., Noto F.K., Sepac A., Sedlic F., Bosnjak Z.J., Lough J.W., Duncan S.A. (2010). Generation of human induced pluripotent stem cells by simple transient transfection of plasmid DNA encoding reprogramming factors. BMC Dev. Biol..

[148] Warren L., Manos P.D., Ahfeldt T., Loh Y.-H., Li H., Lau F., Ebina W., Mandal P.K., Smith Z.D., Meissner A. (2010). Highly efficient reprogramming to pluripotency and directed differentiation of human cells with synthetic modified mRNA. Cell Stem Cell.

[149] Kim D., Kim C.-H., Moon J.-I., Chung Y.-G., Chang M.-Y., Han B.-S., Ko S., Yang E., Cha K.Y., Lanza R. (2009). Generation of human induced pluripotent stem cells by direct delivery of reprogramming proteins. Cell Stem Cell.

[150] Huangfu D., Maehr R., Guo W., Eijkelenboom A., Snitow M., Chen A.E., Melton D.A. (2008). Induction of pluripotent stem cells by defined factors is greatly improved by small-molecule compounds. Nat. Biotechnol..

[151] Muguruma K., Nishiyama A., Ono Y., Miyawaki H., Mizuhara E., Hori S., Kakizuka A., Obata K., Yanagawa Y., Hirano T. (2010). Ontogeny-recapitulating generation and tissue integration of ES cell-derived Purkinje cells. Nat. Neurosci..

[152] Silva T.P., Bekman E.P., Fernandes T.G., Vaz S.H., Rodrigues C.A.V., Diogo M.M., Cabral J.M.S., Carmo-Fonseca M. (2020). Maturation of Human Pluripotent Stem Cell-Derived Cerebellar Neurons in the Absence of Co-culture. Front. Bioeng. Biotechnol..

[153] Hay D.C., Fletcher J., Payne C., Terrace J.D., Gallagher R.C.J., Snoeys J., Black J.R., Wojtacha D., Samuel K., Hannoun Z. (2008). Highly efficient differentiation of hESCs to functional hepatic endoderm requires ActivinA and Wnt3a signaling. Proc. Natl. Acad. Sci. USA.

[154] Toivonen S., Lundin K., Balboa D., Ustinov J., Tamminen K., Palgi J., Trokovic R., Tuuri T., Otonkoski T. (2013). Activin A and Wnt-dependent specification of human definitive endoderm cells. Exp. Cell Res..

[155] Benussi A., Alberici A., Premi E., Bertasi V., Cotelli M.S., Turla M., Dardis A., Zampieri S., Marchina E., Paghera B. (2015). Phenotypic heterogeneity of Niemann-Pick disease type C in monozygotic twins. J. Neurol..

[156] Malnar M., Hecimovic S., Mattsson N., Zetterberg H. (2014). Bidirectional links between Alzheimer’s disease and Niemann-Pick type C disease. Neurobiol. Dis..

